# Spectroscopic Insights into Nutrient-Controlled Biofilm Formation in *Aureobasidium pullulans* as a Basis for Engineered Living Materials

**DOI:** 10.1021/acs.langmuir.6c01932

**Published:** 2026-08-31

**Authors:** Anja Černoša, Jose Manuel Amigo, Fatima Matroodi, Anna Sandak

**Affiliations:** † InnoRenew CoE, Andrej Marušič Institute, 68960 University of Primorska, Muzejski trg 2, SI-6000 Koper, Slovenia; ‡ Department of Analytical Chemistry, 16402 University of the Basque Country UPV/EHU, P.O. Box 644, 48080 Bilbao, Basque Country, Spain; § IKERBASQUE, Basque Foundation for Science, 48011 Bilbao, Spain; ∥ 18474 Elettra Sincrotrone Trieste, Strada Statale 14—km 163.5 in Area Science Park, Basovizza, 34149 Trieste, Italy; ⊥ Faculty of Mathematics, Natural Sciences and Information Technologies, University of Primorska, Glagoljaška 8, 6000 Koper, Slovenia

## Abstract

Fungal biofilms represent complex extracellular systems with potential application in engineered living materials (ELMs), yet their chemical composition and structural variability under defined environmental conditions remain poorly understood. Biofilms of *Aureobasidium pullulans* strains grown on different solid media were investigated using a multimodal spectroscopic approach, including FT-IR, FT-NIR, and Raman spectroscopy. Nutrient-rich media (PDA, SDA, YNB) produced biofilms with largely conserved chemical profiles across strains, whereas nutrient-poor SNA medium induced pronounced changes in polysaccharide, protein, and lipid components, as well as in matrix organization and hydration. Principal component analysis and multiblock data fusion revealed that growth medium is the primary driver of spectral variation, with strain identity exerting a secondary influence. Vibrational bands corresponding to hydroxyl (−OH) and aliphatic (−CH) groups were identified as key contributors to differentiation, reflecting changes in the composition of Extracellular Polymeric Substances (EPS). Raman spectroscopy provided complementary molecular information, such as the presence of characteristic protein and polysaccharide vibrations, which helped confirm the biochemical composition of the biofilms and supported the observations from IR and NIR analyses. Results show that the control of nutrient composition and growth conditions enables precise tuning of biofilm properties, including hydration, matrix density, and functional polysaccharide content. Biofilm composition is primarily influenced by nutrient composition rather than by the strain’s morphological features. This is important because it indicates that research on living coating formulation can focus on supplying nutrients that promote EPS production and biofilm development, rather than on selecting strains with particular morphologies. These findings provide a foundation for understanding the environmental control of *A. pullulans* biofilm formation and are relevant to the future development of engineered living coatings, where environmental conditions can be leveraged to influence biofilm composition, structure, and its functional properties.

## Graphical abstract

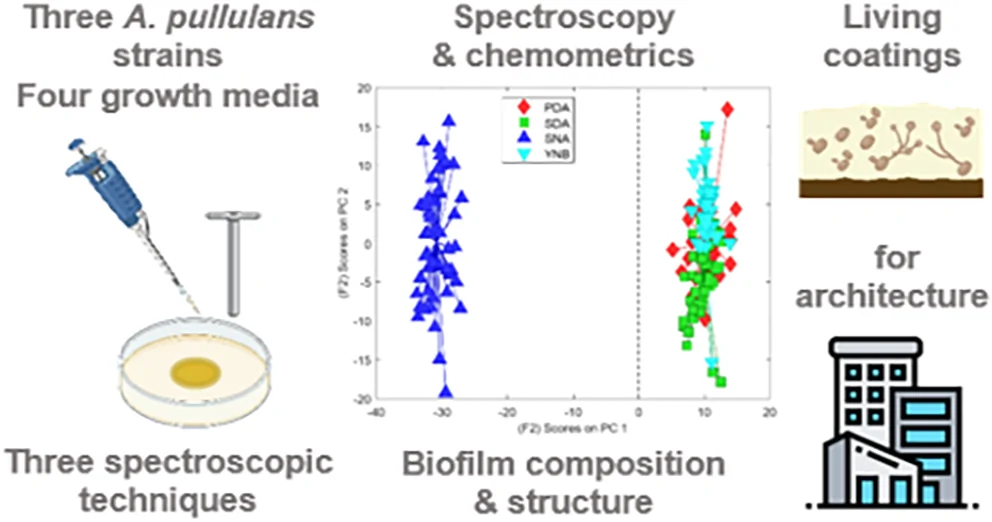

## 1 Introduction

*Aureobasidium pullulans* (de Bary) G. Arnaud is a biotechnologically important black yeast-like fungus.^1,2^ It is polyextremotolerant and therefore occurs in a wide range of environments, from the tropics to the poles, both in nature and in domestic settings.^2^ The fungus *A. pullulans* is often used in biotechnology because of the production of several extracellular enzymes, such as amylases, cellulases, lipases, and proteases, which are useful in various applications.^1^ In addition to these enzymes, *A. pullulans* also produces extracellular polysaccharide pullulan, an important component of the biofilm, which has diverse applications, including food packaging and use as drug capsules.^3,4^ The fungus *A. pullulans* is an attractive candidate for developing Engineered Living Materials (ELMs) for functional surface protection. Its ability to form stable, adaptable biofilms and secrete protective polysaccharides provides a natural basis for self-healing, self-regulating, and environmentally responsive coatings. Within this context, authors explore novel strategies to develop living coatings for architectural applications, where the fungal biofilm is intentionally controlled and optimized to act as a protective, biofunctional layer.^5–7^

Fungal biofilms consist of living fungal cells and an extracellular matrix composed mainly of polysaccharides as well as proteins, lipids, extracellular DNA, and water. This structure enables the cells to adhere to each other and attach to various materials.^8^ The extracellular matrix is also responsible for biofilm architecture.^8,9^ The structure and composition of the biofilm depend strongly on the microorganism and the environmental conditions to which biofilms are constantly adapting.^8^

Fungal biofilms are far less studied than bacterial biofilms, but they have gained increasing attention in recent years. Both yeasts and filamentous fungi can form biofilms. Biofilm development of filamentous fungi involves a few more steps than that of yeasts, which are similar to bacterial biofilm development.^10,11^ Most studies on fungal biofilms have focused on the yeast *Candida albicans* and the filamentous fungus *Aspergillus fumigatus*.^12–15^ The function of the biofilm appears similar to that of bacterial biofilms—it protects the cells from unfavorable external factors and enables them to survive and thrive under extreme environmental conditions.^9,16^ In medicine, where biofilms are most studied, this means that biofilms are more resistant to antifungal drugs than planktonic cells.^8,17,18^

The biofilm produced by *A. pullulans* is mainly studied in agriculture, where *A. pullulans* is used as a biocontrol agent.^19–23^ However, the detailed structure and mechanisms of production are still unknown. This knowledge is essential for our work, as our long-term goal is to develop an active living coating system based on fungal biofilms for the protection of various building materials. Therefore, in this study, we investigated the biofilm formation by three *A. pullulans* strains on different solid media using spectroscopic methods to gain further insight into the structure, chemistry, and development of fungal biofilms.

Fourier transform near-infrared spectroscopy (FT-NIR) and infrared spectroscopy (FT-IR) are powerful analytical techniques that record absorption spectra associated with molecular vibrations of functional groups.^24^ The resulting spectra provide insights into the overall chemical composition and structure of the sample.^25^ FT-IR measures spectra in the range of 400–4000 cm^–1^, whereas FT-NIR covers the region from approximately 4000 to 12000 cm^–1^.^26,27^ FT-IR spectroscopy is more commonly used to study biofilms because it provides high-resolution spectra in the mid-infrared range, which contains strong, well-resolved absorption bands of key biomolecules such as proteins, polysaccharides, and lipids.^24^ The main spectral regions of interest when analyzing biofilms are 3000–2800 cm^–1^ (lipid region), 1800–1500 cm^–1^ (protein region), and 1250–900 cm^–1^ (polysaccharide region).^24^ FT-IR offers several advantages: it is relatively fast and easy to use, requires very little sample, achieves a high signal-to-noise ratio, and allows multivariate statistical analysis of spectral data.^24,26,28^ However, there are also disadvantages. Because biofilms are hydrated, the presence of water may interfere with the spectra. Expertise is required for spectral analysis in chemometrics classification.^26^ FT-IR does not detect elements but only functional groups with dipole momentum, and spectral regions of various components often overlap.^26^ An additional challenge is the lack of available databases and standardized protocols, especially for biological samples.^28^

In contrast, FT-NIR spectroscopy operates at higher wavenumbers, where overtone and combination bands are generally weaker and broader. This makes FT-NIR spectra less straightforward to interpret and, as a result, less commonly applied to biofilm studies, although it offers advantages such as nondestructive analysis and deeper sample penetration.^29–32^

Similar to FT-IR spectroscopy, Raman spectroscopy provides information about molecular vibrations, rotations, and other low-frequency modes in the sample.^16^ Compared with IR spectroscopy, Raman spectroscopy generally uses higher-energy excitation radiation, typically provided by a laser in the near-infrared, visible, or ultraviolet range. Normally, IR signals are stronger than Raman signatures.^33^ However, the advantage of Raman spectroscopy is that its signals are usually not obstructed by water, making it particularly suitable for hydrated biofilms.^33^ Additionally, Raman spectroscopy includes minimal or no sample preparation, nondestructive analysis, high specificity, and minimal interference from water in Raman spectra, making the technique particularly well-suited for analyzing hydrated biofilms.^33,34^ The main disadvantage of Raman spectroscopy is the low signal intensity and noise from electronic, fluorescence, and cosmic sources.^33,35^ Therefore, Raman spectroscopy data must be preprocessed to remove various types of noise and also requires expertise for data analysis.^35^ Deep UV (DUV) Raman spectroscopy is very useful for detecting biochemical compounds because it suppresses the fluorescence, enhancing detection sensitivity and specificity.^36^

Raman and IR spectroscopy are sensitive to different types of chemical bonds. IR is more responsive to polar bonds (e.g., O–H, N–H, C=O), whereas Raman is more sensitive to nonpolar bonds (e.g., C=C, C–C). Therefore, both techniques can provide complementary information on biofilm composition.^16,33^ In addition, Raman microscopy enables high-resolution spatial mapping of chemical heterogeneity within biofilms. Like IR spectroscopy, this method does not require sample preparation and offers nondestructive analysis.^16,37^

While bacterial biofilms have been extensively characterized using various spectroscopic techniques, fungal biofilms remain largely unexplored, with only a few studies reported to date.^15,38–40^ Therefore, this study provides a foundation for further investigation of fungal biofilms using spectroscopic methods. We test the central hypothesis that the chemical composition and spectroscopic signature of *A. pullulans* biofilms are primarily governed by nutrient composition rather than strain-dependent morphological differences under controlled growth conditions. This hypothesis is motivated by the need to identify optimal nutrients and robust environmental control parameters for engineered living material systems, where reproducible modulation of EPS production is essential for functional coating development. FT-NIR, FT-IR, and Raman spectroscopic measurements were performed on biofilms of *A. pullulans* strains exhibiting different morphological growth forms on solid medium. Additionally, the chemical differences of biofilms cultivated on various solid agar media were also analyzed. By integrating FT-NIR, FT-IR, and Raman spectroscopy, this research demonstrates how multimodal analysis can reveal the chemical complexity of *A. pullulans* biofilms under varying conditions. These insights lay the groundwork for designing controlled, functional biofilms as living coatings, advancing the concept of engineered living materials for sustainable architecture.

## 2 Materials and Methods

### 2.1 Strains and Growth Conditions

The strains of *Aureobasidium pullulans* were obtained from Culture Collection Ex of the Infrastructural Centre Mycosmo (Department of Biology, Biotechnical Faculty, University of Ljubljana, Slovenia) (Table 1). The cultures were maintained on malt extract agar (MEA; 5% malt extract agar (Millipore, Burlington, MA, USA) in deionized water) at a temperature of 25 °C.

**Table 1:** List of Strains Used in This Study

culture collection strain number	isolation habitat	geographic location
EXF-3645	glacial ice at the edge of the glacier	Norway
EXF-3844	dried olives	Slovenia
EXF-10507	marble block surface	Italy

### 2.2 Preparation of Biofilm Samples for Spectroscopy Analysis

The strains were inoculated on solid agar medium based on their morphological characteristics. For the yeast-like strain EXF-10507, a cell suspension was prepared from cultures grown on MEA medium in a saline solution to an optical density at 600 nm (OD_600_) of 1.0. Ten microliters of the cell suspension was spotted onto the center of the selected solid medium. For strains EXF-3645 and EXF-3844, which grow filamentously, the solid media were inoculated with 5 mm diameter mycelial plugs from an actively growing colony.

The inoculation was done on four different solid media: defined Yeast Nitrogen Base (YNB) medium (pH 7.0; 0.17% (w/v) yeast nitrogen base (Sigma-Aldrich, Saint Louis, MO, USA), 0.5% ammonium sulfate (Sigma-Aldrich), 2% glucose (Chemlab, Belgium), and 2% agar (Millipore) in deionized water) with a calculated carbon-to-nitrogen (C:N) ratio of 8.8:1; Potato Dextrose Agar (PDA; 3.9% PDA (Millipore)) with estimated C:N ratio = 50:1; Sabouraud solid Agar (SDA; 1% peptone (Millipore), 4% glucose (Chemlab), and 2% agar (Millipore) in deionized water) with calculated C:N ratio = 13.3:1, Synthetic Nutrient deficient Agar (SNA; 0.1% potassium dihydrogen phosphate (Carlo Erba, Italy), 0.1% potassium nitrate (Carlo Erba), 0.05% magnesium sulfate heptahydrate (Carlo Erba), 0.05% potassium chloride (Thermo Fisher Scientific, Waltham, MA, USA), 0.02% glucose (Chemlab), 0.02% sucrose (Sigma-Aldrich), and 2% agar (Millipore) in deionized water) with calculated C:N ratio = 1.18:1. The C:N ratio was calculated from the elemental composition of the carbon- and nitrogen-containing components in the medium. The pH values of the media after autoclaving were 6.3 for YNB, 5.6 for PDA, 5.2 for SDA, and 6.0 for SNA.

Solid media without inoculation were used as controls. The plates were incubated at 25 °C for 14 days to allow the biofilm samples to grow. For each condition, three independent biological replicates were prepared. Each biological replicate consisted of a separately grown culture inoculated onto a single agar plate containing medium.

Because cells are in different metabolic states and at various stages of aging in various regions of the colony,^15^ it was important to always analyze the same part of the colony to avoid spectral heterogeneity caused by these differences. Therefore, measurements were taken approximately half a centimeter from the center of inoculation.

### 2.3 Fourier Transform Near-Infrared Spectroscopy (FT-NIR)

After incubation, near-infrared spectra were measured directly on the biofilms on solid media using an MPA II spectrometer (Bruker Optik GmbH, Ettlingen, Germany), equipped with a fiber-optic probe, operating in the range of 12000 cm^–1^ to 4000 cm^–1^ (833 to 2500 nm). The spectral wavenumber interval was 3.85 cm^–1^, with zero-filling equal to 2. The spectral resolution was 8 cm^–1^, and 32 internal scans were averaged for each spectrum. Three biological replicates of each biofilm sample were prepared, and each sample was subjected to five measurements. For control samples, solid media without inoculated fungi were measured.

### 2.4 Fourier Transform Infrared Spectroscopy (FT-IR)

Infrared spectra were measured using an α II spectrophotometer (Bruker Optik GmbH, Ettlingen, Germany) equipped with a diamond crystal. Spectra were recorded in attenuated total reflectance mode from an average of 64 scans over 4000–400 cm^–1^ at a resolution of 4 cm^–1^. Three biological replicates were prepared for each of the biofilm samples. A piece of each biofilm sample was removed from the plate without any agar attached and placed directly onto the diamond ATR crystal, and its spectrum was recorded. Five spectra were collected from different positions on each sample. For control samples, a piece of solid medium was taken from the plate and placed directly on a diamond ATR crystal.

### 2.5 Deep Ultraviolet Raman Scattering (DUVR) Spectroscopy

Deep Ultraviolet Raman scattering (DUVR) spectroscopy was used directly on biofilms grown on solid media, with three biological replicates per condition. For control samples, solid media without inoculated fungi were measured directly. Deep UV Raman spectra were collected at the IUVS laboratory of Elettra Sincrotrone Trieste (Italy) using a portable Photon Systems Inc. PL-200 Raman microscope featuring a 248.6 nm NeCu gas laser. The excitation source operated at an average pulse energy of 6 μJ and a repetition rate of 40 Hz. The spectrometer was configured with a 3600 g/mm grating, providing a spectral resolution of <25 cm^–1^ over the spectral range of 400–3700 cm^–1^. A 5× objective lens was used to focus the excitation beam onto the sample and to collect the backscattered Raman signal. The system was calibrated using the known Raman shifts of spectroscopic-grade acetonitrile (Sigma-Aldrich) as the external standard. Spectra were recorded with a 2.5 s acquisition time per point. For each sample, measurements were taken at three distinct locations within the central area of the Petri dish, with 66 points mapped at each location, yielding a total of 198 spectra per sample for subsequent analysis.

### 2.6 Analysis of Spectroscopic Data

Spectral preprocessing methods included standard normal variate (SNV), first derivative, and multiblock scaling for data fusion. Spectral preprocessing and principal component analysis (individual spectroscopies and data fusion) were performed using Opus 6.5 (Bruker, Ettlingen, Germany) and PLS_Toolbox (eigenvector Inc., Manson, IA, USA), an extension of the MATLAB package (MathWorks Inc., Natick, MA, USA). The spectral interpretation was performed according to the bands presented in Table 2. The individual spectra collected from the same biological sample were not treated as independent biological observations in the ASCA model but served as technical replicates, which were used to assess local spectral variability and measurement repeatability. For statistical modeling, spectra were averaged at the biological replicate level before ASCA was performed. Therefore, the effective number of independent observations corresponded to the biological replicates, not to the individual spectral acquisitions, to avoid inflation of statistical significance due to pseudoreplication.

**Table 2:** Most Significant Bands Obtained in the Types of FT-NIR, FT-IR and Raman Spectroscopy Vibration with Particular Assigned Components

wavenumber (cm^–1^)	assignment and the type of vibration	remarks
FT-IR		
900–600		fingerprint region peaks^24,41^
∼1040	stretching vibration of C–O–C group, C–O stretching of β-glucan	polysaccharides, nucleic acids^42^
1180	C–O–C asymmetric stretching of glycosidic linkage, C–O stretching of sugars	polysaccharides, nucleic acids^42^
1540	combination of N–H bending and C–N stretching	amide II
1650	C=O stretching vibrations	amide I
2930	alkane C–H stretching^24,41,43^	lipids^42,44,45^
NIR		
5200	OH stretching and HOH deformation modes of polysaccharides^27^	carbohydrate^27^
6000	2 C–H str.^46^	carbohydrates, lipids^46^
6900	first overtone of the OH-stretching^47^	water^47^
7250	2 C–H str. + C–H def^46^	carbohydrates, lipids^46^
Raman		
1000	ammonium sulfate^48^	
1060	potassium nitrate^49,50^	
1250	amide III^51^	lipids, proteins^44^
1390	δ (COH), (HCO), (HCC), ν_s_ (COO−), (C–O)^44^	polyanionic polysaccharide^44^
1490	cis amide II	lipids, proteins^44^
1530	C=C	DNA bases^44^
1650	C=O stretching, OH bending mode of pyranose group, and OH bending mode of water	amide I, water^51,52^

## 3 Results

### 3.1 Growth of Biofilm Samples

Fungal growth started at the central inoculation point and expanded radially during incubation, forming a circular colony. As observed in Figure 1, the biofilms were thinner on the SNA medium than on the other media, indicating differences in colony morphology under various growth conditions.

**Figure 1:**
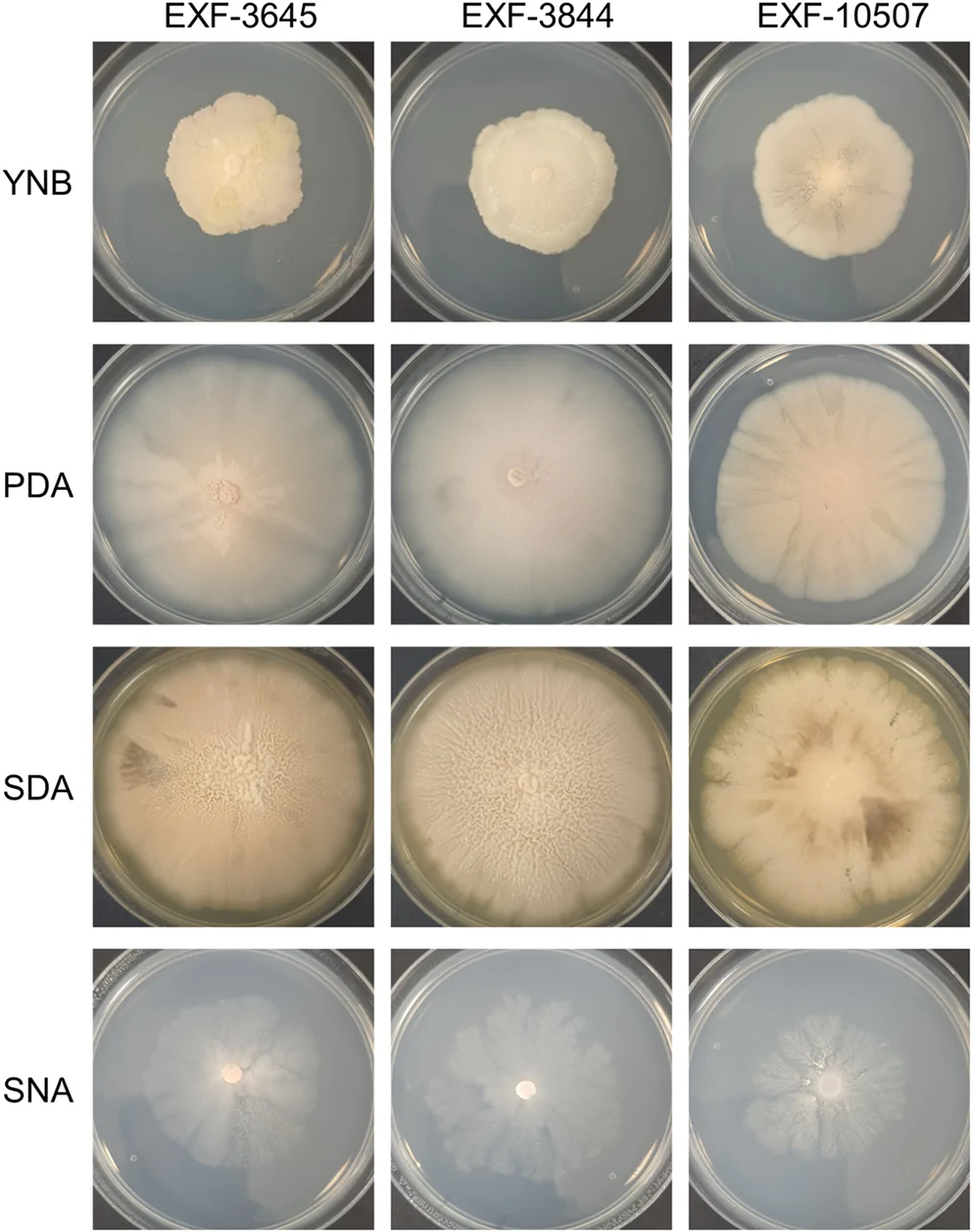
Growth of *Aureobasidium pullulans* biofilms of strains EXF-3645, EXF-3844, and EXF-10507 on four solid media (YNB, PDA, SDA, SNA) after 14 days of incubation at 25 °C.

The architecture of biofilms depends on both the strain and medium. All tested *A. pullulans* strains grow on all four media, but growth on the SNA medium is more filamentous and weaker compared with the other three media. Strain EXF-10507 grows more in the yeast form, while strains EXF-3645 and EXF-3844 grow more in the filamentous form, which is most visible on the SDA medium.

### 3.2 FT-NIR Results

#### 3.2.1 Initial Observations or Raw and Preprocessed Data

The raw NIR data already reveal clear structural patterns (Figures S1 and S2). Notably, the control samples are grouped closely, indicating an internal consistency. In contrast, the biofilms grown on the SNA medium appear divided into three subgroups, although this separation may result from scattering effects. A similar trend is observed in the biofilms grown on the YNB medium, particularly with one specific strain (EXF-10507), suggesting potential variability within the same class.

After applying smoothing and SNV, stronger patterns emerge (Figures S3 and S4). Two major groups appear, indicating that the medium has a greater influence than the strains. This suggests that medium conditions are a stronger source of variability than the biology of the strains.

#### 3.2.2 Principal Component Analysis

To further investigate the patterns observed in the NIR data, principal component analysis (PCA) was performed (Figure 2). The PCA model clarified the sources of variability among the biofilm samples. Along PC1, a clear distinction is evident between biofilms grown on SNA medium and control samples versus the other groups, highlighting the pronounced effect of nutrient composition. PC2 further separates biofilms grown on SNA medium from the control samples, revealing more subtle differences between these two groups. Interpreting the loading vectors is somewhat complex; however, strong contributions from O–H functional groups, likely originating from water and sugar components, are evident. Overall, the PCA confirms that, while the medium exerts a dominant influence on the biofilm spectra, intrinsic differences between biofilms grown on SNA medium and control samples are also detectable.

**Figure 2:**
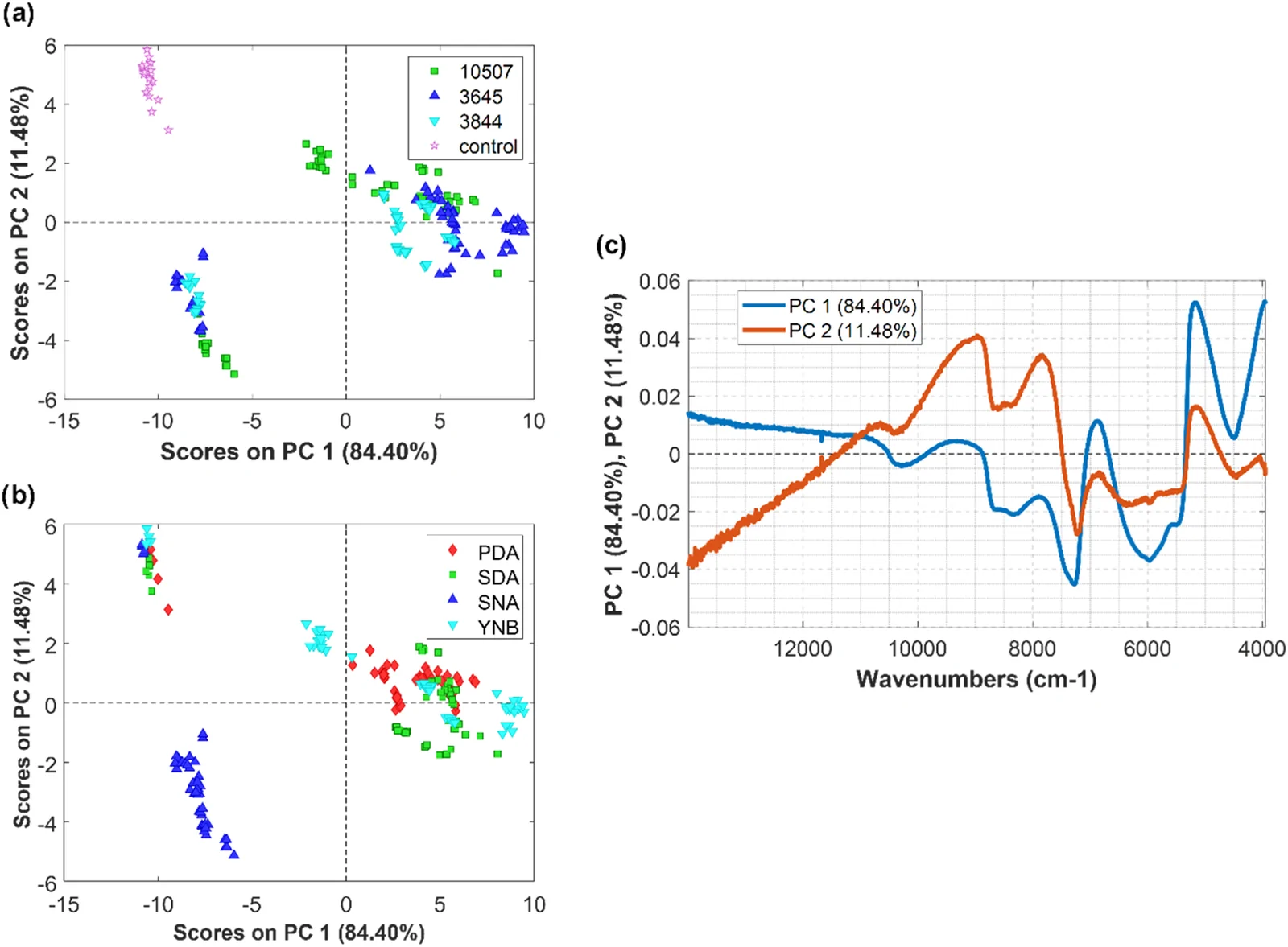
Principal component analysis of NIR data based on (a) strain and (b) medium. Graph (c) represents the loadings of PCA NIR spectra.

#### 3.2.3 PCA with First Derivative

Application of PCA to the first-derivative NIR data provides additional insight into the spectral variability among the biofilm samples (Figure 3). In the PC1-PC4 scores, biofilms grown on the SNA medium are more distinctly separated from the other groups, indicating that derivative preprocessing enhances spectral features specific to this medium and its associated biofilm composition. Biofilms grown on the YNB medium remain divided into two subclusters, confirming that internal heterogeneity within this class is not an artifact of baseline effects but reflects genuine biochemical or morphological variation. The use of PC4, rather than PC2, improves the visualization of these differences and highlights additional sources of variability that are not captured in the lower components.

**Figure 3:**
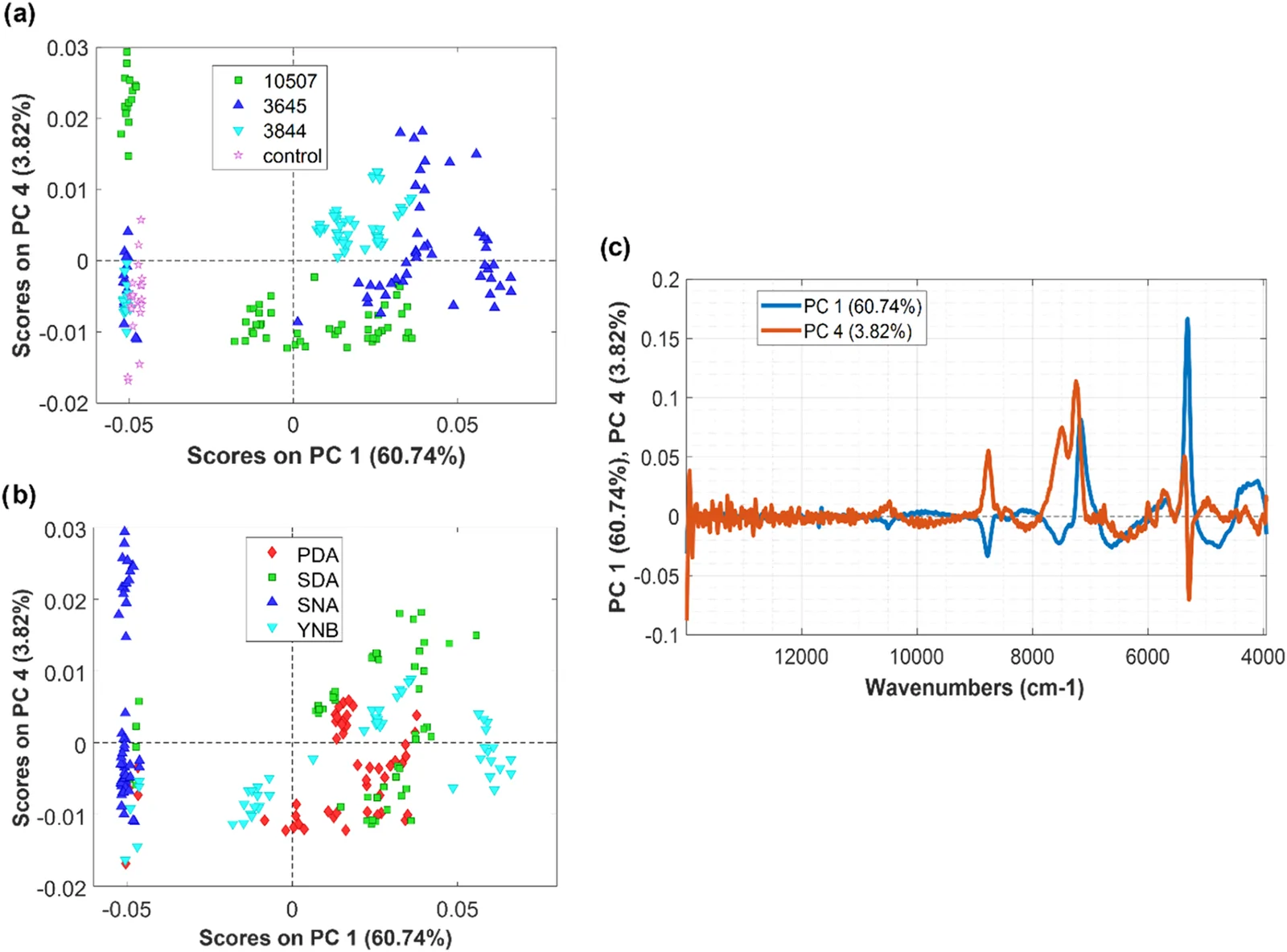
PCA with first derivative based on (a) strain and (b) medium. Graph (c) represents a loadings plot of PCA with the first derivative of NIR spectra.

The corresponding loading plot highlights the spectral regions responsible for these separations. A prominent derivative feature around 7000 cm^–1^, associated with O–H and N–H overtone bands, suggests that differences in water–matrix interactions or hydration patterns, influenced by both fungal activity and medium composition, are key drivers of the observed clustering. Additional contributions near 5400 cm^–1^ (the O–H combination band) further support the interpretation that variations in moisture content and water-binding environments play a major role. The PCA results show that the main sources of spectral variability reflect not only biological differences between fungal strains but also substantial medium-dependent effects. In fact, the medium appears to exert a stronger influence on the NIR signatures, likely through differences in water structure, nutrient composition, and biofilm hydration, than the difference between strains alone.

### 3.3 FT-IR Results

#### 3.3.1 Initial Observations or Raw and Preprocessed Data

Analysis of the IR data revealed one clear outlier among the samples. Aside from this, several patterns were apparent (Figures S5, and S6). A shift toward lower wavenumbers, around 500 cm^–1^, appears to correspond with the growth medium, suggesting that the medium composition influences specific molecular vibrations. The spectra also display a complex, peak-rich region near 1000 cm^–1^, corresponding to the classic fingerprint region and reflecting the diverse chemical composition of the biofilms. These preliminary observations set the stage for further exploration using multivariate analyses.

After SNV preprocessing, previous trends become more apparent (Figures S7 and S8). The simplicity of IR preprocessing is advantageous here as patterns emerge clearly without complex transformations. The clear outlier is the biofilm of strain EXF-10507 grown on SNA medium, the fourth replication, which is associated with a specific medium (SNA).

#### 3.3.2 PCA on Preprocessed IR Data

After removing the outlier, PCA of the IR data reveals more detailed patterns than those seen in the NIR spectra (Figure 4). The PC1 versus PC2 scores plot shows a strong separation between most biofilms grown on SNA and YNB media, with biofilms grown on SNA medium clearly distinct and positioned near the control samples. Examination of the loading vectors highlights key vibrational regions, particularly around 500 cm^–1^ and 1000 cm^–1^. These regions, rich in sugar-associated peaks, are likely to be especially informative if modeled separately. Overall, the observed separation reflects not only the biological class but also the influence of the growth medium, highlighting the combined effect of these factors on the IR spectral profiles.

**Figure 4:**
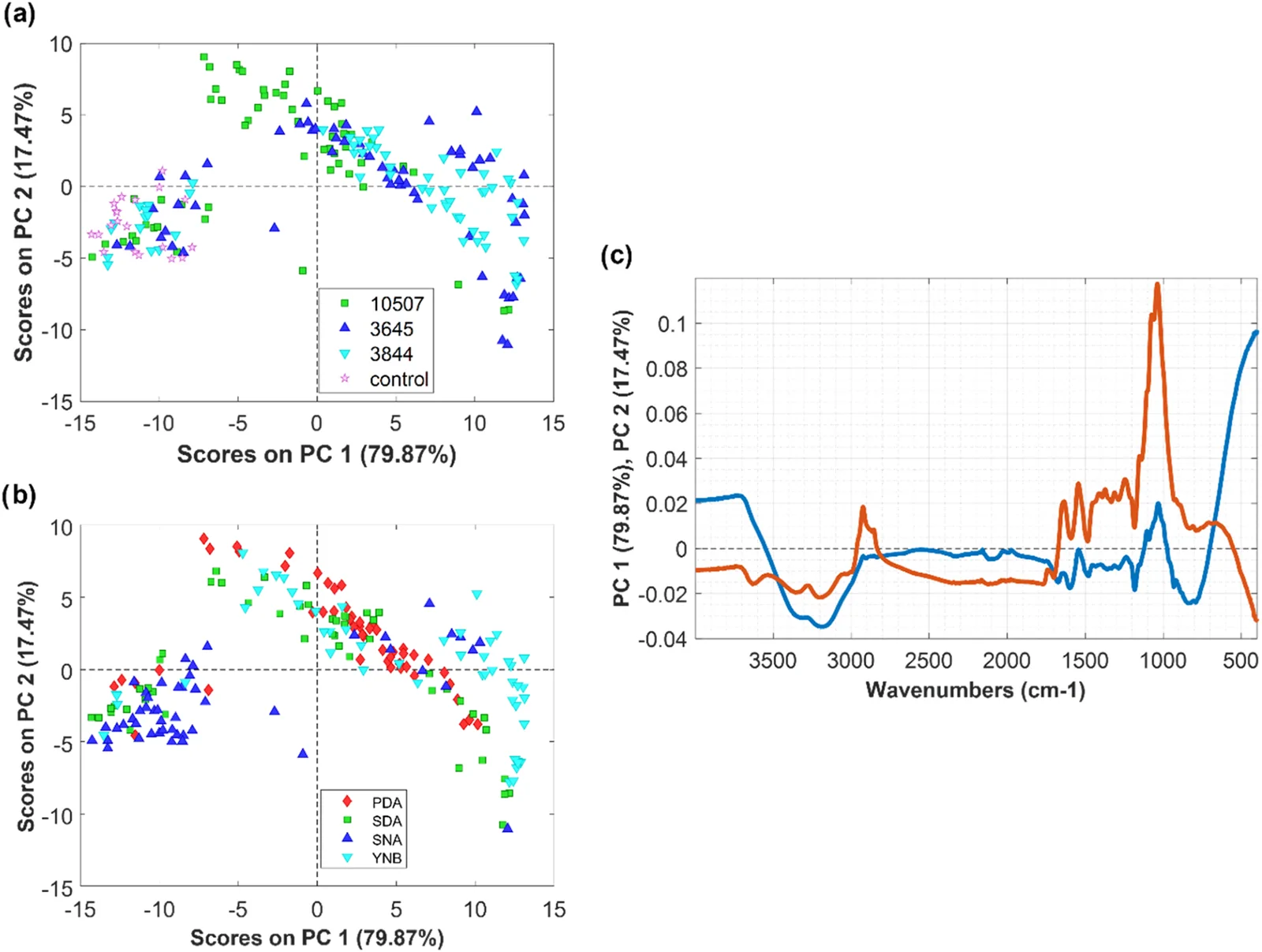
Principal component analysis of FT-IR data based on (a) strain and (b) medium. Graph (c) represents the loadings of PCA FT-IR spectra.

### 3.4 Multiblock Data Fusion

#### 3.4.1 Head: Simplest Data Fusion Merged NIR with IR and Further PCA

To explore the combined information from both spectral ranges, a simple data fusion approach was used by merging first-derivative NIR data with SNV-processed IR data (Figure 5). After fusion, block-variance scaling was applied to balance the contributions from each data set.

**Figure 5:**
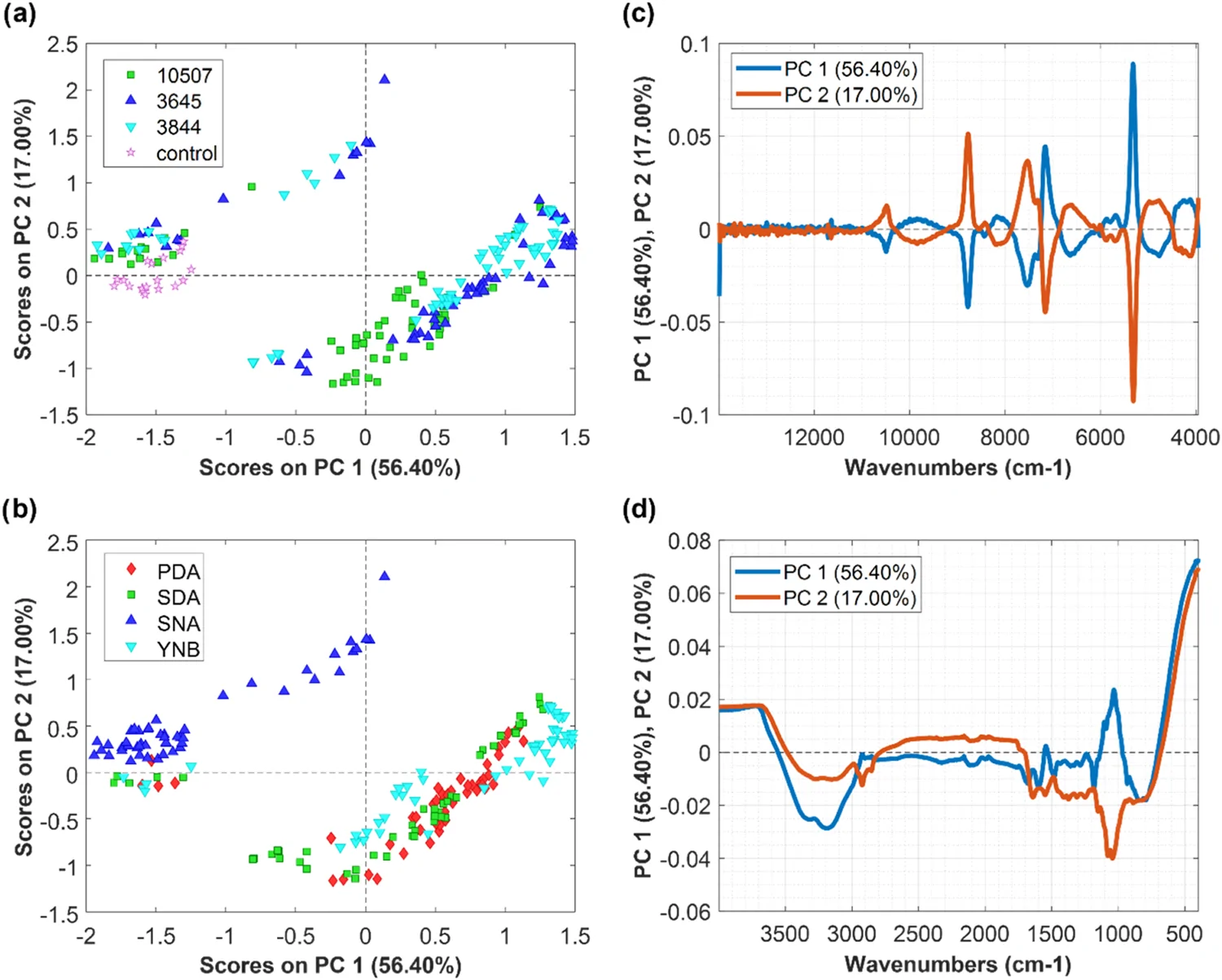
Simple data fusion of NIR and FT-IR data based on (a) strain and (b) medium. Graphs represent the loadings of PCA (c) FT-NIR and (d) FT-IR spectra.

The fused data set largely reinforces the patterns observed in the individual analyses. Notably, the effects previously captured along PC2 in the separate models now appear along PC3, reflecting a redistribution of the explained variance across the principal components. Despite this shift, the influence of the growth medium remains the dominant factor structuring the data, confirming that the medium composition strongly drives variability in the biofilm spectra even when NIR and IR information are combined.

#### 3.4.2 Multiblock ASCA Results

The multiblock ASCA model offers an additional layer of insight into the factors driving the variability in the biofilm spectra (Figure 6). Factors 1 and 2, corresponding to growth medium and strain, respectively, are highly significant (*p* = 0.001) and produce clear separations among the sample groups in the scores space. These results highlight the combined influence of both the medium composition and biological strain on the spectral profiles, reinforcing the patterns observed in the PCA analyses.

**Figure 6:**
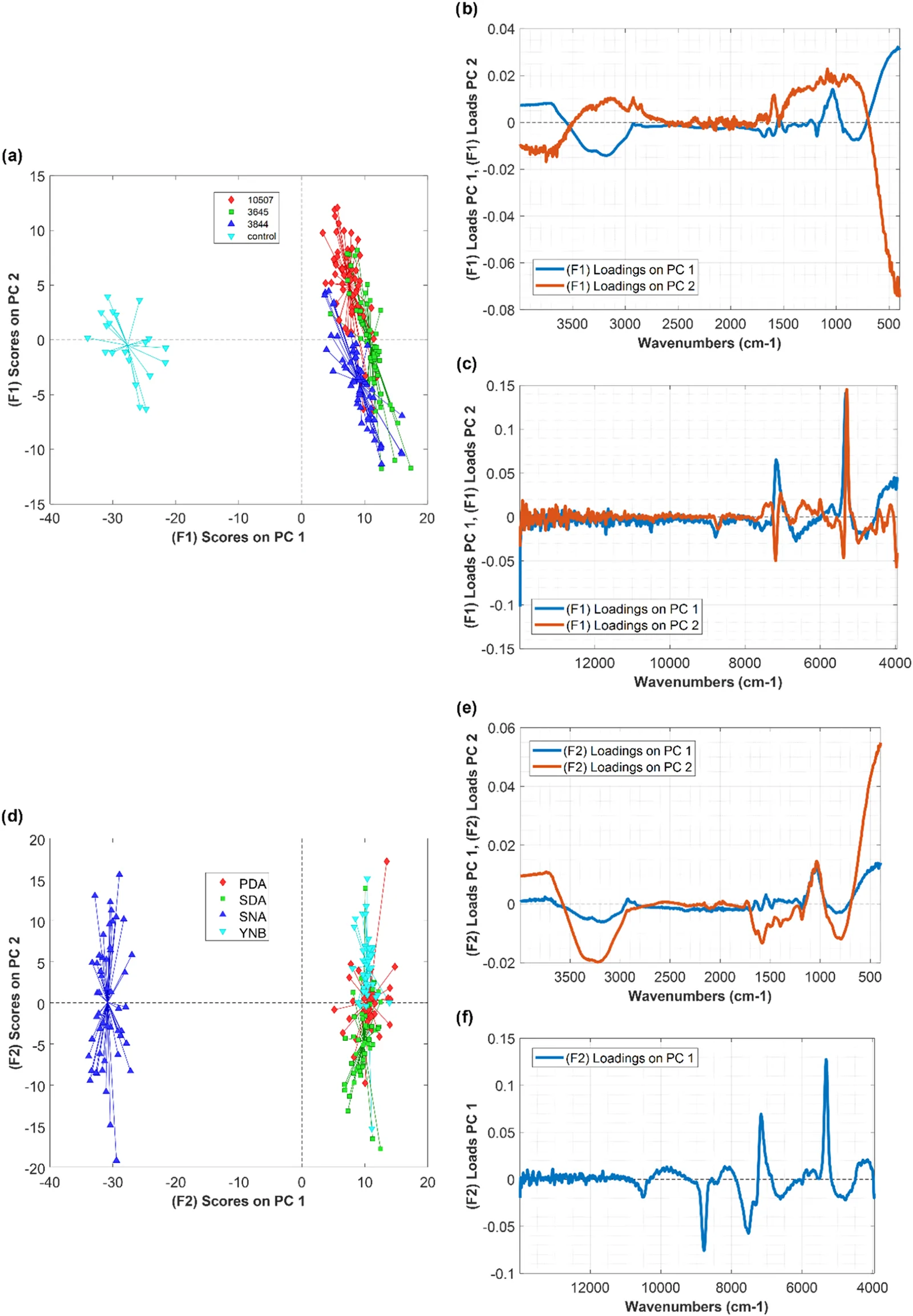
Multiblock ASCA results based on strain (a–c) and media (d–f).

Additionally, a major insight, as shown in Figure 6, is the benefit of the multiblock scenario: while 2 PCs are somewhat important for IR, only 1 PC in NIR is linked to both variabilities (media and strain).

#### 3.4.3 Combined Effect

When factors such as strain in the multiblock ASCA model are combined, strong separation among the sample groups is maintained (*p* = 0.001) (Figure 7). Individual groups, such as biofilms of strain EXF-10507 grown on the PDA medium or biofilms of strain EXF-3645 grown on the SDA medium, are clearly distinguished in the score space. Notably, the most informative wavenumber regions are consistent across both IR and NIR data sets, reinforcing the conclusion that the medium is a primary driver of spectral variation.

**Figure 7:**
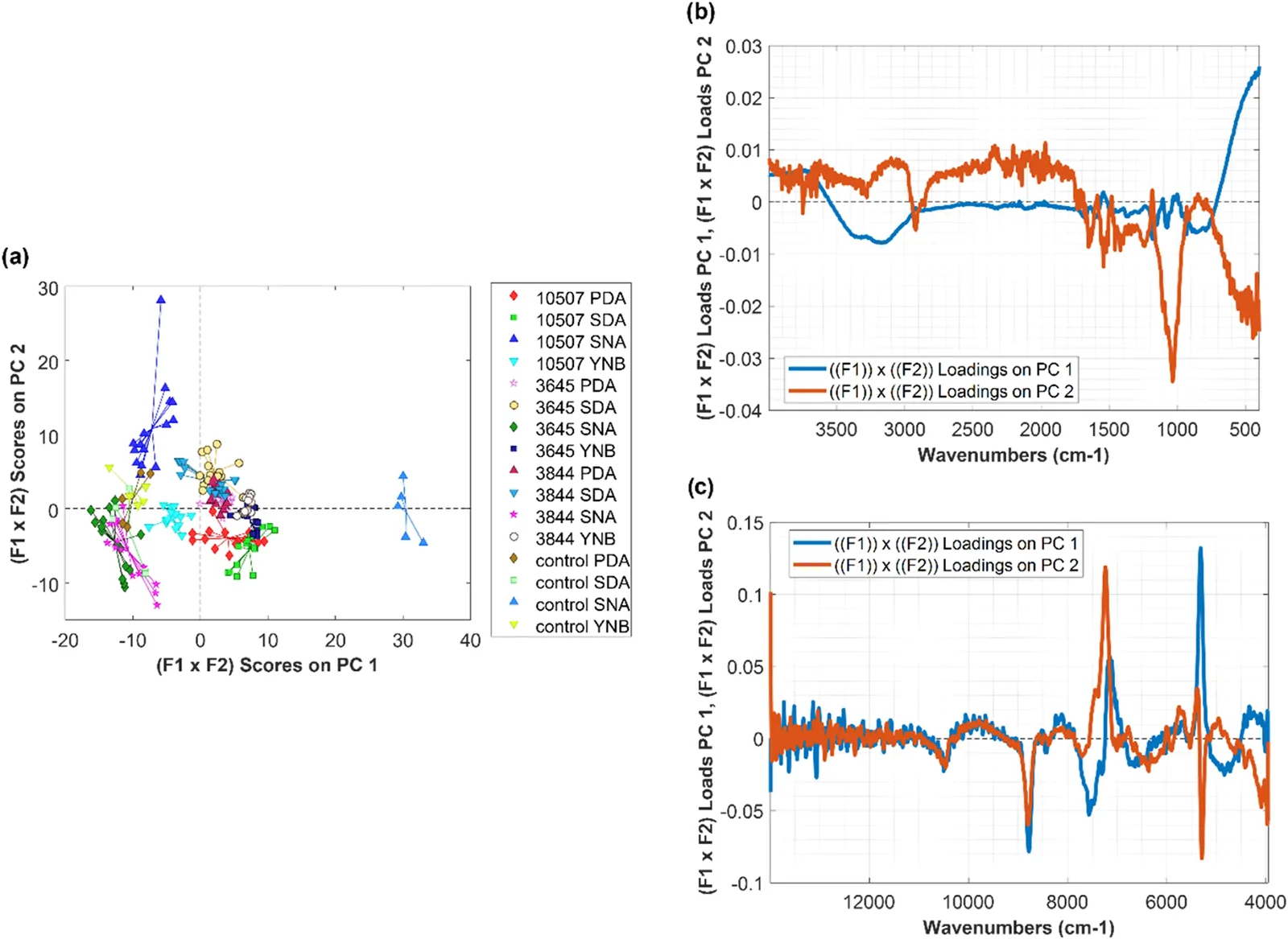
(a) Combined effect of both FT-IR and FT-NIR, (b) loadings of FT-IR interactions, and (c) loadings of FT-NIR interactions.

FT-IR spectroscopy provides detailed information, capturing characteristic features across multiple biochemical regions. In the fingerprint region, relative intensities reveal distinct patterns for the lipid region (3000–2800 cm,^–1^), the amides I and II region (1700–1470 cm^–1^), and the carbohydrate region (1200–700 cm^–1^). These features underscore the higher information content of FT-IR spectra compared to FT-NIR, highlighting the technique’s sensitivity to key biofilm components.

### 3.5 Deep Ultraviolet Raman scattering (DUVR) Spectroscopy Results

#### 3.5.1 Initial Observations or Raw and Preprocessed Data

By utilizing an excitation wavelength below 250 nm, fluorescence is significantly minimized, leaving the fingerprint region virtually fluorescence-free, and allowing the detection of characteristic vibrational bands associated with biofilm biomolecules (Figure 8). The DUVR Raman spectra were first averaged for each sample to reduce noise and spectral variability (Figures S9, and S10). The Raman spectra of the control groups exhibit a prominent water contribution in the 3000–3700 cm^–1^ region, which is particularly pronounced in SNA and YNB media. Each control group displays distinct Raman features that allow for clear differentiation. For instance, SNA exhibits a sharp band around 1060 cm^–1^ and a relatively stronger band at 1650 cm^–1^ compared to the other groups. Conversely, PDA medium shows distinguishing bands around 1250, 1340, and 1490 cm^–1^. In the case of YNB medium, the characteristic bands are located around 1000 and 1580 cm^–1^. The strong band around 1060 cm^–1^ in the SNA medium spectrum is likely associated with potassium nitrate.^49,50^ Similarly, the band appearing near 1000 cm^–1^ in the YNB medium spectrum may be associated with ammonium sulfate.^48^

**Figure 8:**
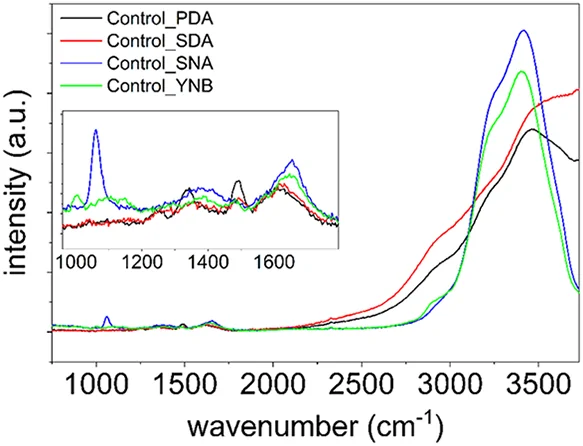
Mean Raman spectra of control groups, averaged by group.

#### 3.5.2 PCA on Preprocessed Raman Data

PCA performed on the full Raman data set initially limited the interpretability of the scores (Figure 9). To address this, the analysis was restricted to the spectral interval between 600 and 2200 cm^–1^, a promising region for capturing relevant molecular features. To enhance spectral interpretability, baseline correction using weighted least-squares was applied as part of the preprocessing workflow.

**Figure 9:**
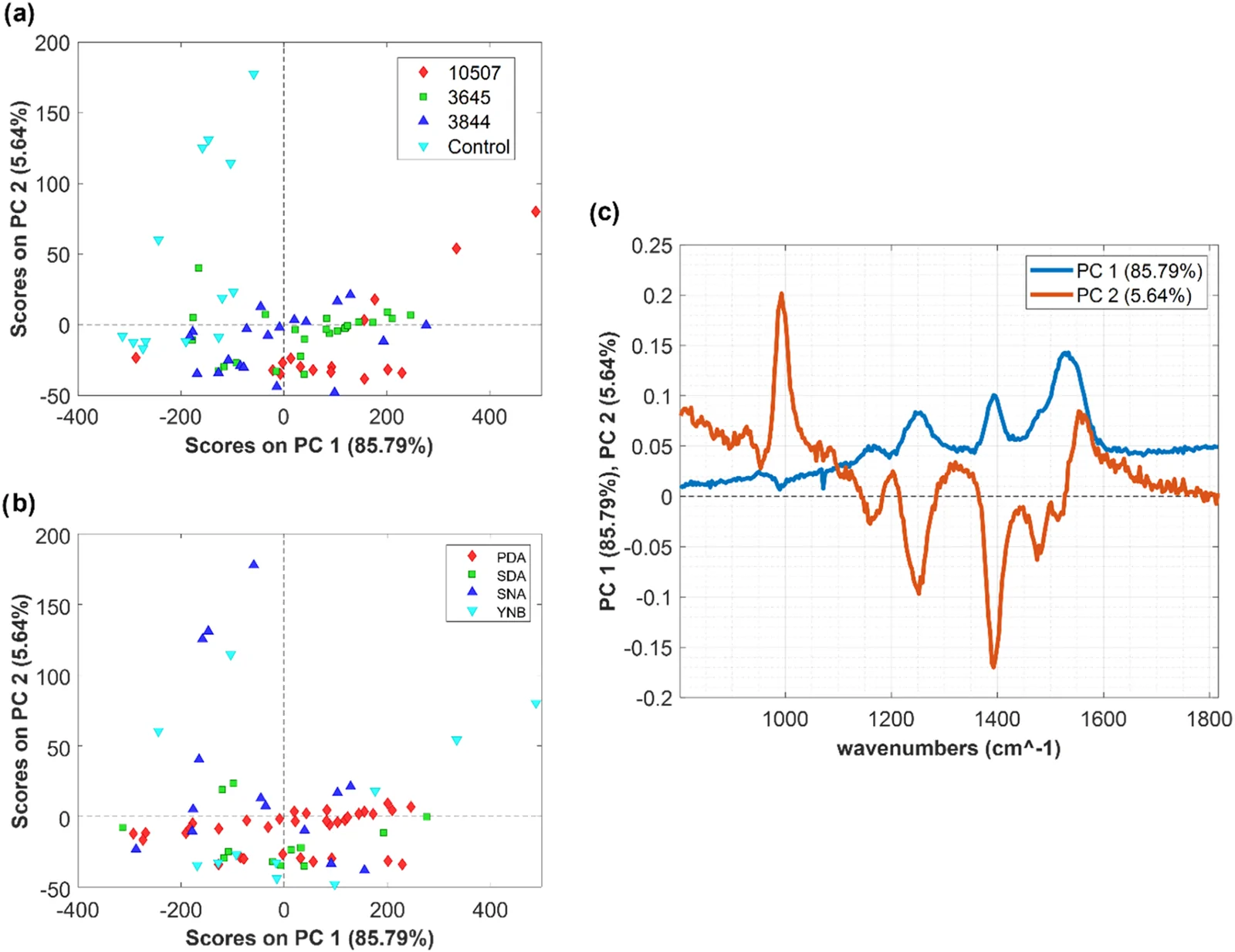
Principal component analysis of DUV Raman spectroscopy data based on (a) strain and (b) medium. (c) Loadings of PCA DUV Raman spectroscopy spectra.

Despite this adjustment and after testing various preprocessing strategies, including derivatives, a clear separation of sample groups – such as that observed in the NIR and IR data sets—remains elusive. While the restricted interval highlights key peaks, further optimization of preprocessing methods is necessary to fully exploit the discriminative potential of the Raman spectra.

## 4 Discussion

The fungus *Aureobasidium pullulans* is recognized for its importance in various biotechnological applications and is receiving increasing attention as a potential candidate for producing biobased coatings in the field of Engineered Living Materials (ELMs).^1,2,6,53^ However, its biofilm structure and composition remain largely unknown. In this study, we analyze the composition and structure of *A. pullulans* biofilm using three spectroscopic methods—FT-NIR, FT-IR, and Raman spectroscopy—to conduct a multimodal analysis.

Three morphologically distinct strains of *A. pullulans* were selected to determine whether different morphologies affect the biofilm chemical composition. The distinct morphologies of the strains were observed while growing on the MEA solid medium: strain EXF-3844 displayed a filamentous growth pattern, strain EXF-10507 exhibited a yeast-like growth form, and strain EXF-3645 grew in both yeast and filamentous forms. These differences were consistent across the replicate plates and incubation periods. Additionally, four different solid media were chosen to examine how nutrients affect biofilm structure, as it was already reported in the literature that external conditions influence biofilm formation.^8^ Three nutrient-rich media (PDA, SDA, and YNB) with different C:N ratios and one nutrient-poor medium (SNA) were selected. As shown by macroscopic observations (Figure 1), the biofilms differ more due to nutrient composition than between the strains. Macroscopic observations also reveal contrasting characteristics between nutrient-rich and nutrient-poor media.

To investigate the chemical differences in biofilms between different strains and between biofilms grown on different media, we used Fourier transform near-infrared spectroscopy (FT-NIR), infrared spectroscopy (FT-IR), and DUV Raman spectroscopy. All three techniques are complementary, as they cover a wide range of absorbance from 400 cm^–1^ to 12000 cm^–1^.^26,27^ Additionally, they access different regions of vibrational energy and show varying sensitivities to functional groups.^16,24,33^ Preprocessing spectra obtained from all three techniques is necessary to reduce noise or unwanted background information and enhance the signal.^35,54^ Therefore, replicative measurements of the same sample are required to lower noise levels.^54^ Preprocessing is also necessary for all spectra to correct variations associated with spectral acquisition.^28,54^

Preprocessed data can then be analyzed using various analytical tools, enabling more in-depth analysis and detection of subtle differences in absorbance patterns.^24^ The most commonly used multivariate method for spectral differentiation is principal component analysis (PCA), an unsupervised dimensionality-reduction technique that transforms correlated spectral variables into a smaller set of orthogonal principal components, capturing the maximum variance in the data and facilitating visualization and pattern recognition.^54^

The biofilm samples in this study contain the entire colony biomass, including intact cells and extracellular polymeric substances (EPS). Therefore, the signals from spectroscopic measurements are attributed to both the EPS and the cell wall compositions. It should be noted that the biofilm samples were not subjected to EPS extraction or purification prior to spectroscopic measurements. Therefore, the spectral bands discussed in relation to EPS composition cannot be attributed exclusively to the extracellular matrix. While complementary biochemical assays, such as phenol–sulfuric acid and protein quantification methods, could provide additional information on the relative abundance of polysaccharides and proteins, such analyses were beyond the scope of the present study. Future work combining spectroscopic measurements with biochemical characterization of isolated EPS fractions would help further validate and refine the spectral assignments.

FT-IR and FT-NIR spectral data showed that despite morphological differences among *A. pullulans* strains, the overall chemical composition of their biofilms was similar under nutrient-rich conditions (PDA, SDA, YNB). This indicates that the biofilm matrix composition is relatively conserved among different strains under favorable environments. A similar pattern was observed in biofilms of the yeast *Brettanomyces bruxellensis*, where spectra from different strains showed little distinction.^38^ A substantial spectral shift was detected in biofilms grown on nutrient-poor SNA medium, indicating that the chemical composition of the biofilm responds to the nutrient composition. The observed spectral differences on the SNA medium may also reflect changes in the physical organization of the biofilm, including thickness, hydration, or density of the matrix. Hydration, in particular, strongly affects the −OH stretching region, explaining some of the pronounced features observed in the NIR spectra. Similarly, changes in the spectra of the yeast *Candida albicans* grown on media with different dextrose concentrations were detected,^15^ which is also consistent with the phenotypic analysis of *C. albicans* biofilms grown on different types of media.^55^ Raman spectra highlight specific vibrational features that may provide additional chemical insights, particularly for components that are less accessible in the IR and NIR spectra. In this study, the deep-UV Raman approach effectively minimized fluorescence in the fingerprint region compared with conventional Raman measurements, improving spectral quality. Nevertheless, the observed spectral differences between groups were limited, and multivariate preprocessing did not yield distinct clustering. Consequently, the Raman results should be interpreted as supporting the presence of characteristic biofilm biomolecular components rather than providing definitive evidence of compositional differences among the groups. Additional spectral analysis and validation would be required to establish such differences with greater confidence.

To support our findings, a literature search was conducted to assign the type of vibration and compounds to selected groups of wavenumbers (Table 2). Loadings plots from PCA showed that the vibrational bands corresponding to the −OH groups (around 5200 cm^–1^ and 7250 cm^–1^ in FT-NIR) and the −CH groups (around 1000 cm^–1^ in FT-IR) contribute most strongly to the differentiation of the samples.^27,41,46^ The strong −OH signals likely originate from polysaccharide components such as pullulan, which is rich in hydroxyl groups.^4^ The −CH signals indicate changes in aliphatic (lipid or protein) components of the biofilm matrix.^26,42,44,45^

The spectral trends observed in the IR fingerprint region (around 400–1500 cm^–1^) are also present in the overtone and combination bands of the NIR spectra (especially between 4000–7500 cm^–1^). This parallel suggests that subtle structural or compositional changes in the biofilm are detectable by both direct molecular vibrations (IR) and their harmonic combinations (NIR). Since all strains are reported to grow in filamentous form on SNA, the observed spectral differences could reflect not only changes in chemical composition but also in physical organization, such as thickness, hydration state, or density of the biofilm matrix. In particular, hydration strongly affects the −OH stretching region present in the NIR spectra. The strong contribution of −OH-related spectral bands indicates that hydration effects may substantially influence the observed spectral differences between biofilms. Because water is a major component of the biofilm matrix, variations in moisture content, water–matrix interactions, biofilm thickness, and biomass density may contribute to the separation observed in both IR and NIR analyses. Consistent with this interpretation, the control samples exhibited more pronounced water-associated bands in the high-frequency region of the Raman spectra. Therefore, the observed spectral differences should be interpreted as reflecting a combination of hydration state, physical organization, and biochemical composition. Future studies incorporating direct measurements of water content, biomass, colony thickness, and EPS composition would help to further separate these contributions.

Importantly, prior to fusion, block-variance scaling was applied to each spectroscopic data set to balance their contributions and avoid bias toward modalities with higher variance or dimensionality. Under these conditions, FT-IR contributed detailed information related to specific biochemical constituents, FT-NIR captured changes associated with hydration and matrix organization, and Raman spectroscopy provided complementary information on vibrational modes that are less affected by water. Thus, the value of the multimodal approach lies not only in improved discrimination but also in obtaining a more comprehensive biochemical description of the biofilm composition and structure. ASCA integrates the principles of ANOVA with multivariate decomposition, enabling the partitioning of spectral variability into contributions that can be attributed to specific experimental factors and their interactions. This method is particularly useful for the investigation of biofilm spectroscopy as it facilitates the identification of biologically meaningful variation while minimizing confounding effects from correlated spectral features. Data fusion and multiblock ASCA models confirmed the reproducibility of these patterns and highlighted key wavenumber regions that consistently differentiated samples. These results reinforce the notion that environmental conditions, particularly nutrient composition, play a critical role in shaping the biofilm chemistry and structure.

Fungus *A. pullulans* responds to various environmental conditions, including nutrient composition. The nutrient composition of the growth medium affects the ratio of filamentous to yeast-like cells and the production of extracellular polysaccharides, which directly influence spectroscopy measurements.^56–58^ However, there is limited knowledge about the relevance of *A. pullulans* morphological plasticity^57^ and which cell type produces more extracellular polysaccharides. Most existing knowledge about fungal dimorphism is based on fungal pathogens, where the transition from yeast cells to hyphae is important for host invasion and virulence.^59^

Overall, the combined spectroscopic analyses show that both the chemical composition and structural organization of *A. pullulans* biofilms are sensitive to the environmental conditions. Nutrient-poor media, such as SNA medium, induce detectable changes in polysaccharide content, protein, and lipid composition and matrix organization, while nutrient-rich conditions maintain a conserved biofilm profile across strains. These findings have direct implications for the development of engineered living coatings, as controlling nutrient composition and growth conditions could enable precise tuning of biofilm properties including hydration, matrix density, and functional polysaccharide content. It provides insights into the biofilm composition and structure of the fungus *A. pullulans* and establishes foundational knowledge for preparing living coatings based on a fungal biofilm. Since biofilm formation is largely influenced by nutrient availability and composition, coating formulation strategies can prioritize supplying nutrients that promote EPS production and biofilm development rather than focusing solely on selecting strains with specific morphological traits. This perspective is particularly relevant for predictive maintenance approaches, where controlled nutrient supplementation during service life could be used to sustain or enhance biofilm growth and functionality over time.

The interpretations presented here are based primarily on the spectroscopic signatures and multivariate analysis. Although the observed spectral variations are consistent with differences in polysaccharide-, protein-, lipid-, and water-associated components of the biofilms, the present study does not provide direct quantitative measurements of these constituents. Additional analyses, including biomass determination, water content measurements, colony thickness assessment, and biochemical characterization of EPS components, would provide further validation of the proposed compositional differences and should be considered in future studies. It is important to note that the present study employs simplified agar-based growth systems as a controlled model for isolating the influence of nutrient composition on biofilm formation. While these conditions do not fully replicate the complexity of real building environments, they provide a necessary baseline for separating the key biochemical drivers of biofilm development. In practical applications such as building protection, *A. pullulans* biofilms would form on mineral and organic substrates under fluctuating environmental conditions, including humidity cycles, UV exposure, and temperature variations. Nevertheless, nutrient composition is expected to remain a dominant factor influencing EPS production and matrix composition, particularly during the early colonization and establishment phases. Therefore, the trends observed here provide insights that can inform future research under more complex, application-relevant conditions. Future studies will examine the relationships between the biochemical composition and performance in real-world applications, such as the spectroscopy of biofilms grown on various types of building materials. Additionally, biofilms used in field testing are exposed to varying environmental conditions such as temperature fluctuations, reduced water availability, and exposure to UV radiation and light. Obtaining more information about biofilm composition and structure in these experiments would also provide further insight into *A. pullulans* biofilms in natural environments. Future work should also aim to quantitatively correlate specific spectroscopic features with functional properties such as mechanical stability, adhesion, and water retention, enabling the prediction of living coating performance and linking it to spectral data. Complementary biochemical and microscopic analyses would further validate spectral interpretations and provide deeper mechanistic insights into biofilm matrix organization. Nevertheless, by combining three spectroscopic techniques (FT-IR, FT-NIR, and Raman spectroscopy), three *A. pullulans* strains, and four growth media, this study provides a systematic framework for evaluating how strain and environmental conditions influence fungal biofilm formation and its structural and chemical characteristics, setting a foundation for the rational design of functional, sustainable, and adaptive surface coatings.

## 5 Conclusions

This study demonstrates that the chemical composition and structural organization of *A. pullulans* biofilms are strongly influenced by the growth medium, while strain identity plays a secondary role under nutrient-rich conditions. FT-IR and FT-NIR spectroscopy revealed that nutrient-rich media support a conserved biofilm matrix across strains, whereas nutrient-poor conditions (SNA) induce pronounced changes in polysaccharide, protein, and lipid components as well as in matrix organization and hydration. Raman spectroscopy provided complementary information regarding the biochemical composition of the biofilms. Its capacity to distinguish among the four media highlights its potential for a further detailed characterization. Multimodal analysis, including PCA, data fusion, and multiblock ASCA, confirmed that medium composition is the dominant driver of spectral variation and that specific wavenumber regions, particularly those associated with −OH and −CH vibrations, are informative for distinguishing biofilm states. The combined use of FT-IR, FT-NIR, and Raman spectroscopies provides a comprehensive view of biofilm chemical and structural properties, capturing both detailed functional group information and bulk matrix characteristics. The value of the multimodal approach, besides improving discrimination, provided in this case a comprehensive biochemical description of the biofilm composition and structure.

This study provides foundational spectroscopic and chemometric characterization of *A. pullulans* biofilms under controlled environmental conditions. While functional coating performance has not yet been evaluated, the results establish a basis for the future development of engineered living coatings and their application in architecture. In particular, the observed ability to modulate EPS composition and matrix structure through environmental control highlights a promising route toward active biobased surface systems. Future work will extend these findings to different substrates and real-world environmental conditions, where adhesion, durability, and protective functionality can be directly assessed, further clarifying how controlled variation in nutrient composition influences biofilm composition, EPS production, and biofilm matrix organization.

## Supplementary Material



## Notes

The right description would be: Raw and preprocessed spectra from FT-NIR measurements based on the strain; raw and preprocessed spectra from FT-NIR measurements based on the medium; raw and preprocessed spectra from FT-IR measurements based on the strain; raw and preprocessed spectra from FT-IR measurements based on the medium; raw and preprocessed spectra from Raman spectroscopy measurements based on the strain; raw and preprocessed spectra from Raman spectroscopy measurements based on the medium (Figures S1-S10) (PDF)

## Abbreviations

ELMs: engineered living materials
FT-NIR: Fourier transform near-infrared spectroscopy
FT-IR: Fourier transform infrared spectroscopy
DUV Raman spectroscopy: deep UV Raman spectroscopy
YNB: yeast nitrogen base medium
PDA: potato dextrose Agar
SDA: Sabouraud solid Agar
SNA: synthetic nutrient deficient Agar
SNV: standard normal variate
PCA: principal component analysis
ATR: attenuated total reflectance
DUVR spectroscopy: deep ultraviolet Raman scattering spectroscopy
