# Supplementary material for: Spectroscopic Insights into Nutrient-Controlled Biofilm Formation in Aureobasidium pullulans as a Basis for Engineered Living Materials
Source: Langmuir. 2026 Aug 31;42(36):26144–57. doi: 10.1021/acs.langmuir.6c01932 (PMC13585130; doi:10.1021/acs.langmuir.6c01932)
Supplement: Supplementary file 1 [file la6c01932_si_001.pdf]

Supporting information

Spectroscopic insight into nutrient-controlled biofilm formation in *Aureobasidium pullulans* as a basis for engineered living materials

Anja Černoša[1,], Jose Manuel Amigo[2,3], Fatima Matroodi[4], Anna Sandak[1,5]*

[1] InnoRenew CoE, Andrej Marušič Institute, University of Primorska, Muzejski trg 2, SI-6000 Koper, Slovenia

[2] Department of Analytical Chemistry, University of the Basque Country UPV/EHU, 48080, Bilbao, P.O. Box 644, Basque Country, Spain

[3] IKERBASQUE, Basque Foundation for Science, 48011 Bilbao, Spain

[4] Elettra Sincrotrone Trieste, Strada Statale 14 - km 163.5 in AREA Science Park, Basovizza, 34149, Trieste, Italy

[5] Faculty of Mathematics, Natural Sciences and Information Technologies, University of Primorska, Glagoljaška 8, 6000 Koper, Slovenia

Corresponding author: Anja Černoša (anja.cernosa@innorenew.eu)

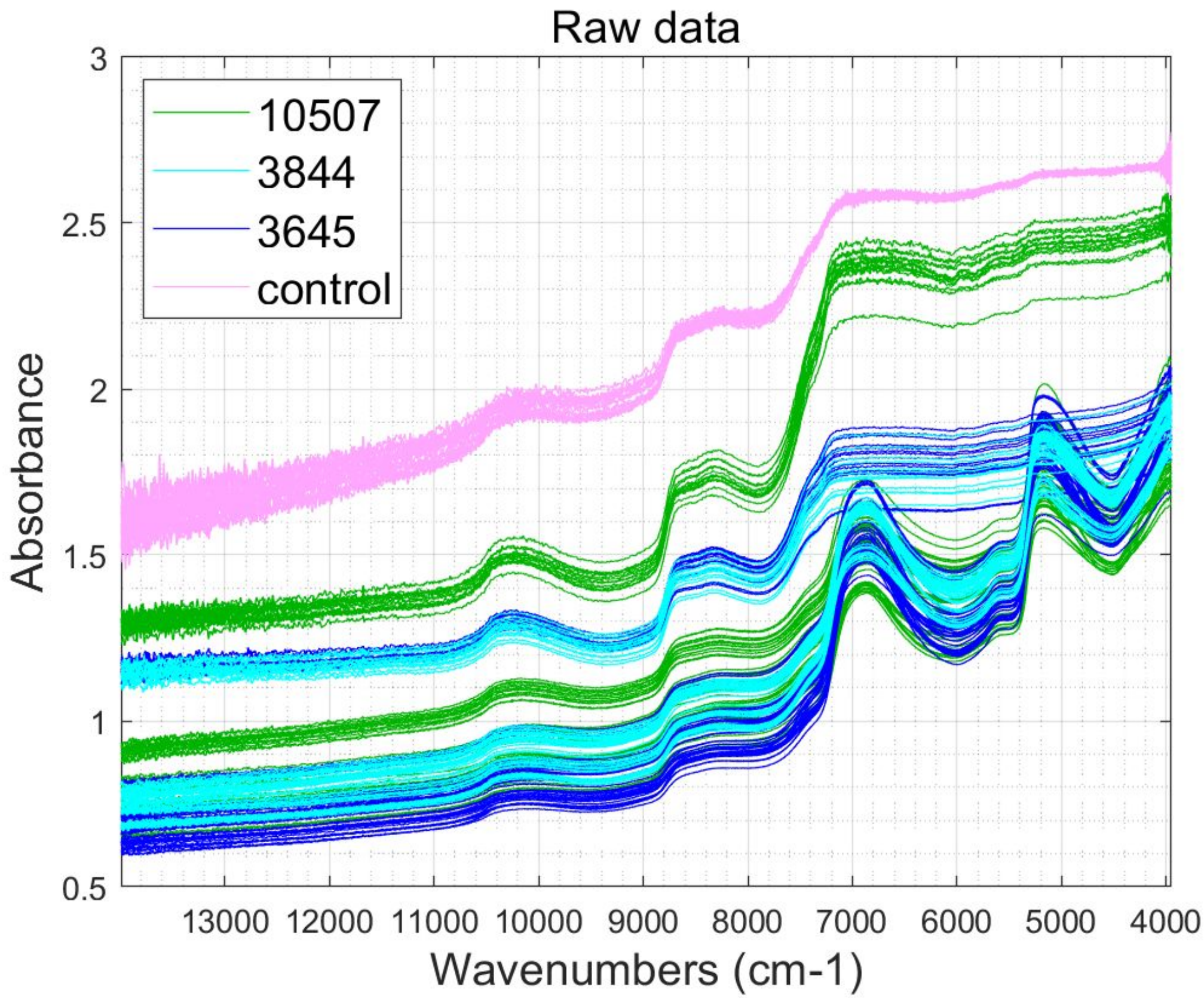

Figure S1: Raw spectra from NIR measurements based on strain.

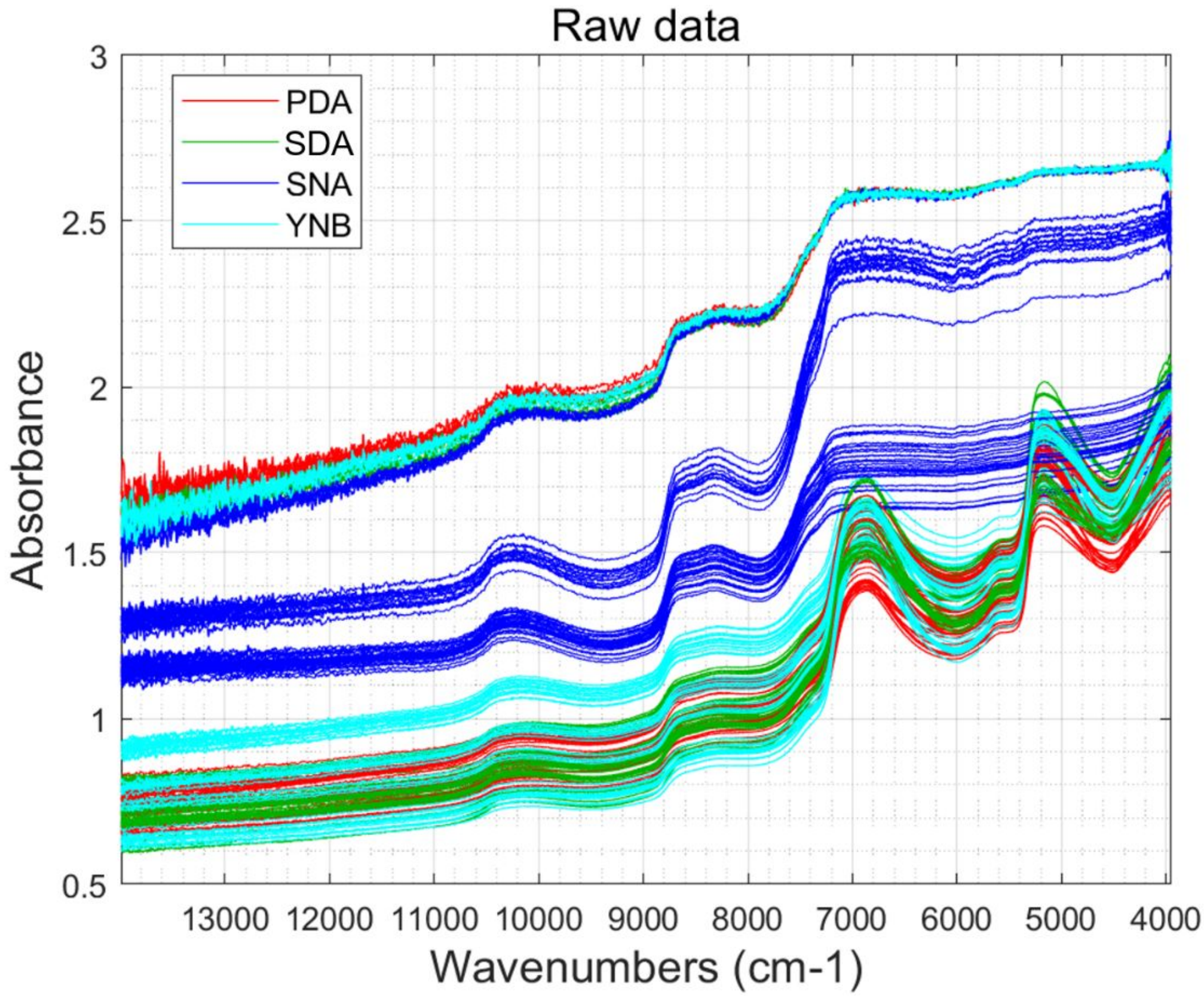

Figure S2: Raw spectra from NIR measurements based on the medium.

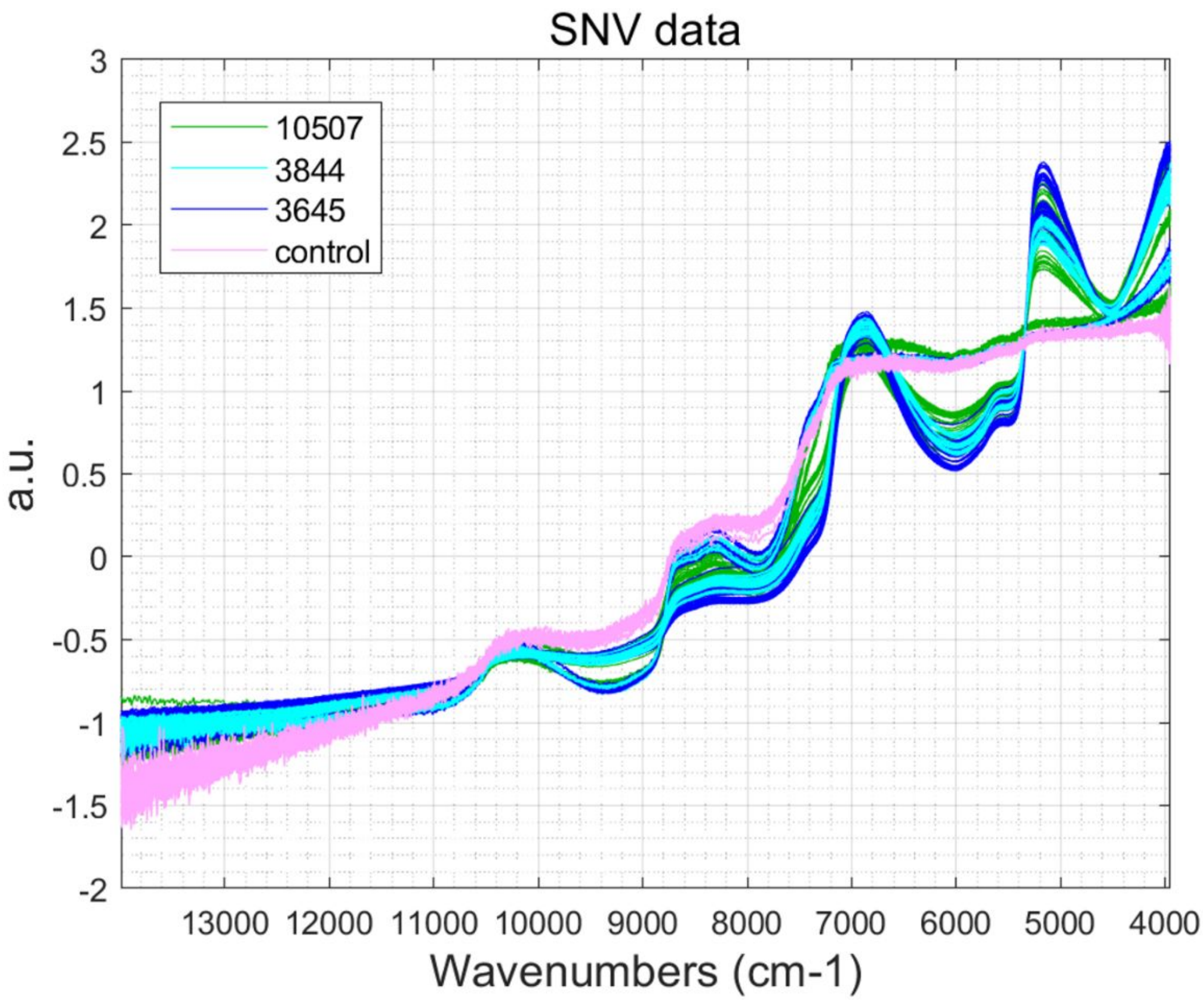

Figure S3: Preprocessed spectra from NIR measurements based on strain.

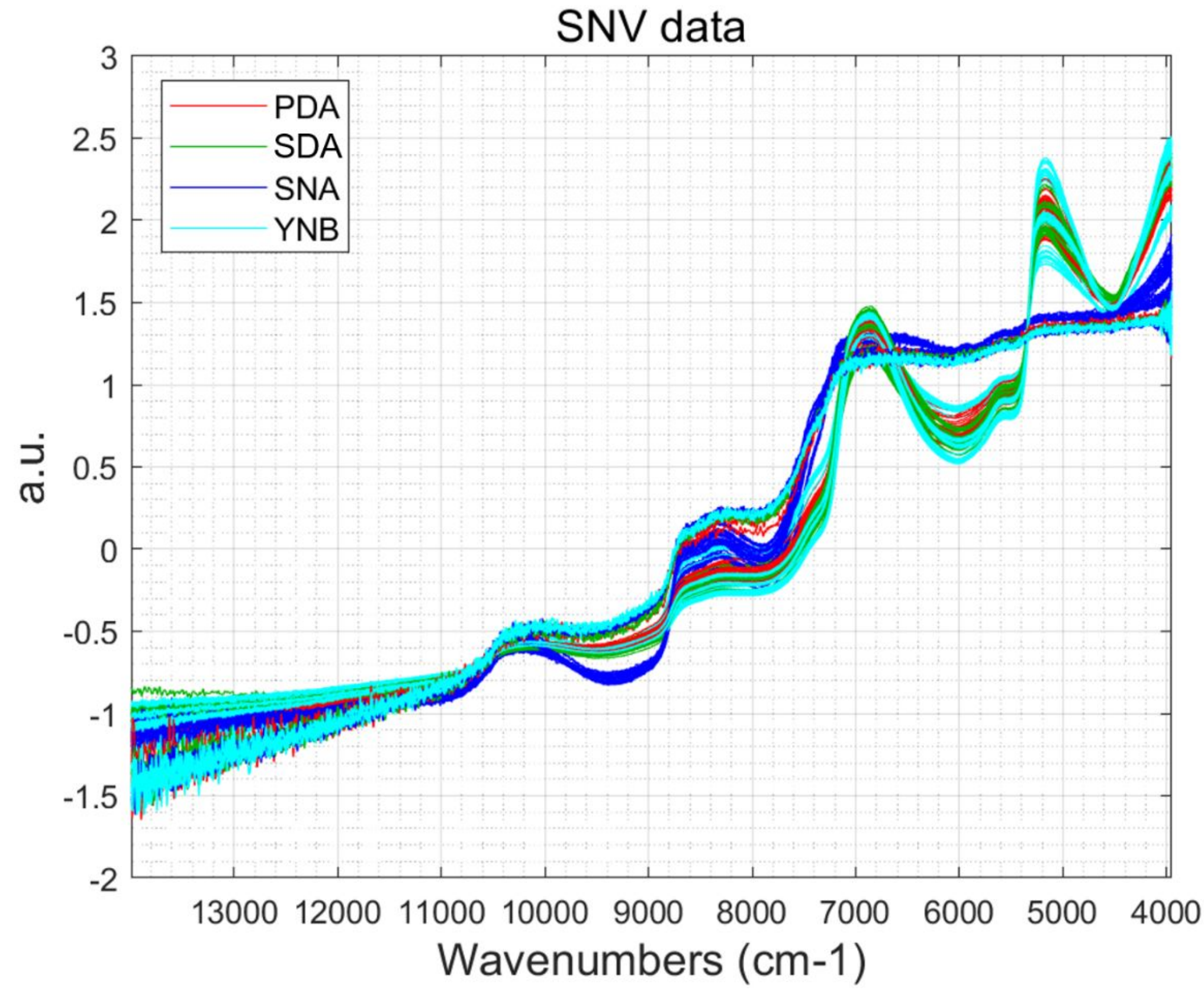

Figure S4: Preprocessed spectra from NIR measurements based on the medium.

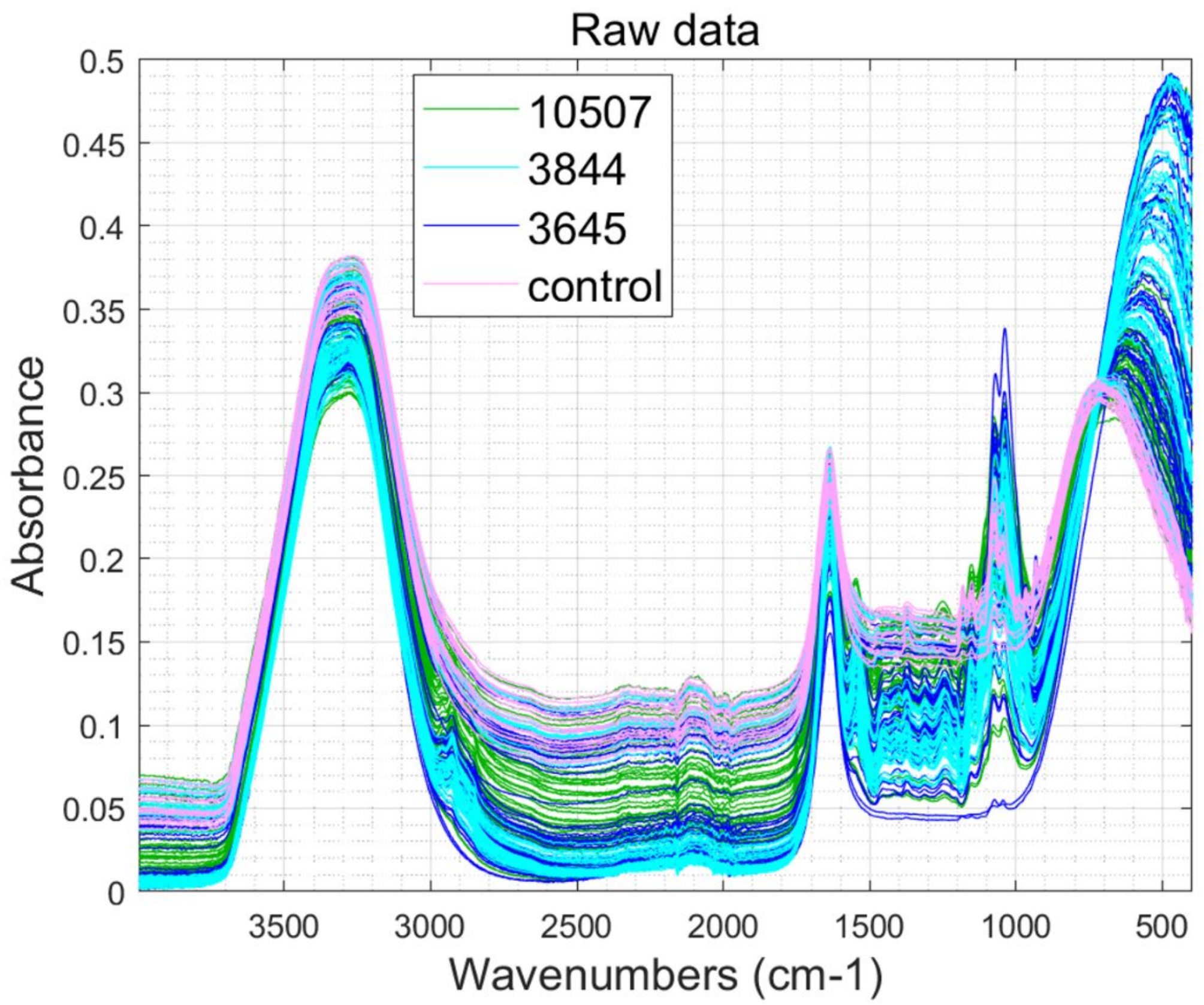

Figure S5: Raw spectra from FT-IR measurements based on strain.

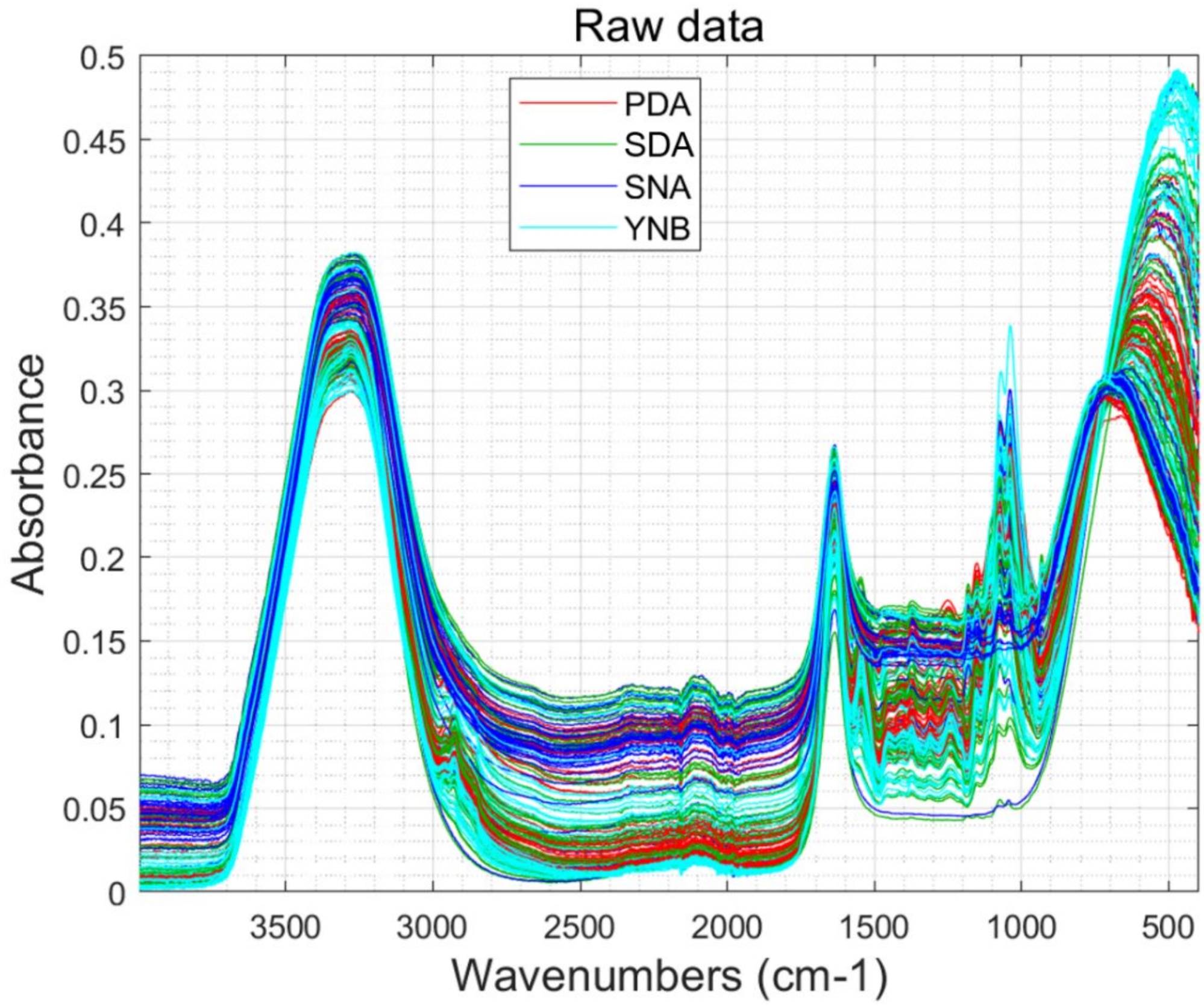

Figure S6: Raw spectra from FT-IR measurements based on the medium.

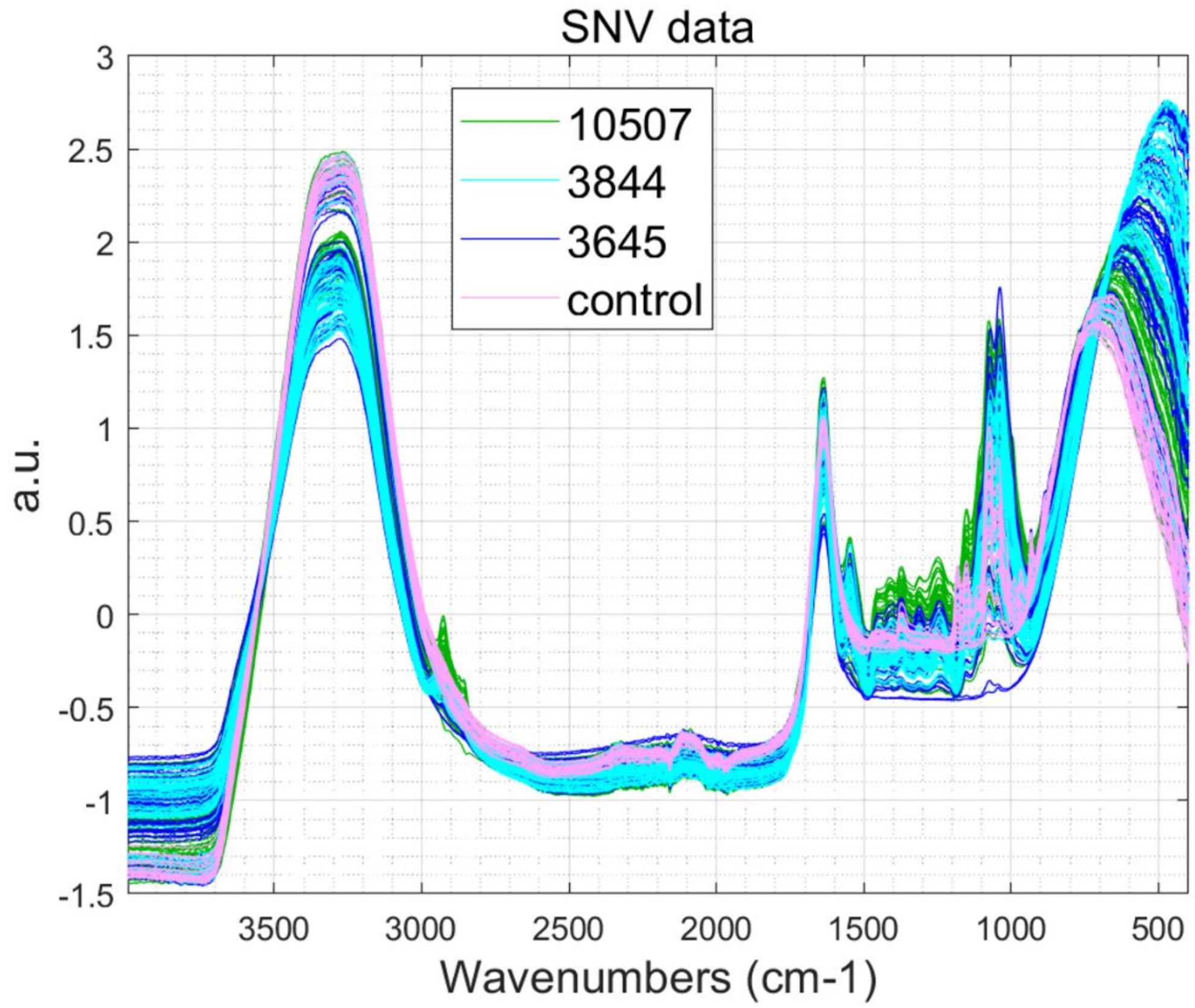

Figure S7: Preprocessed spectra from FT-IR measurements based on strain.

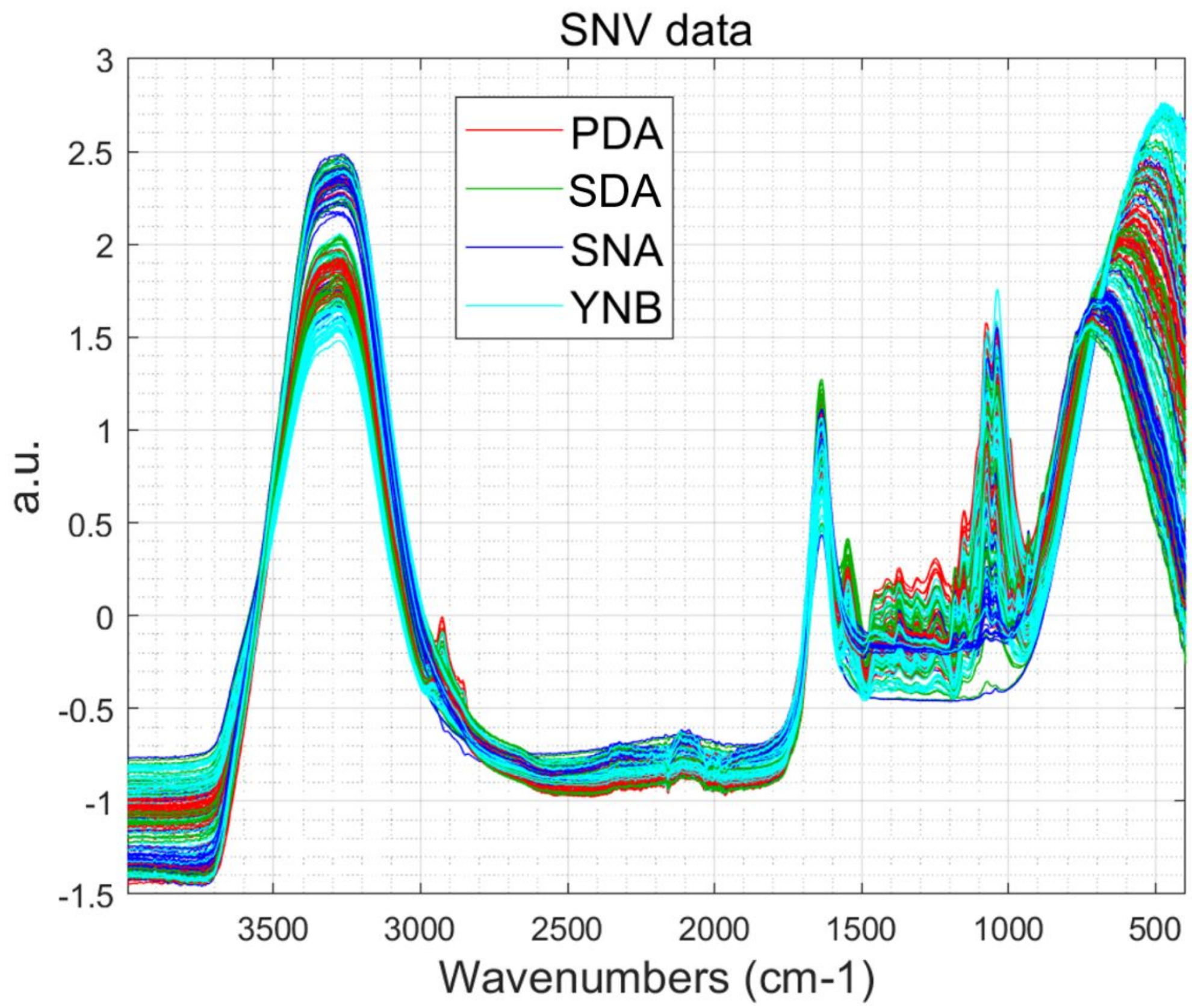

Figure S8: Preprocessed spectra from FT-IR measurements based on the medium.

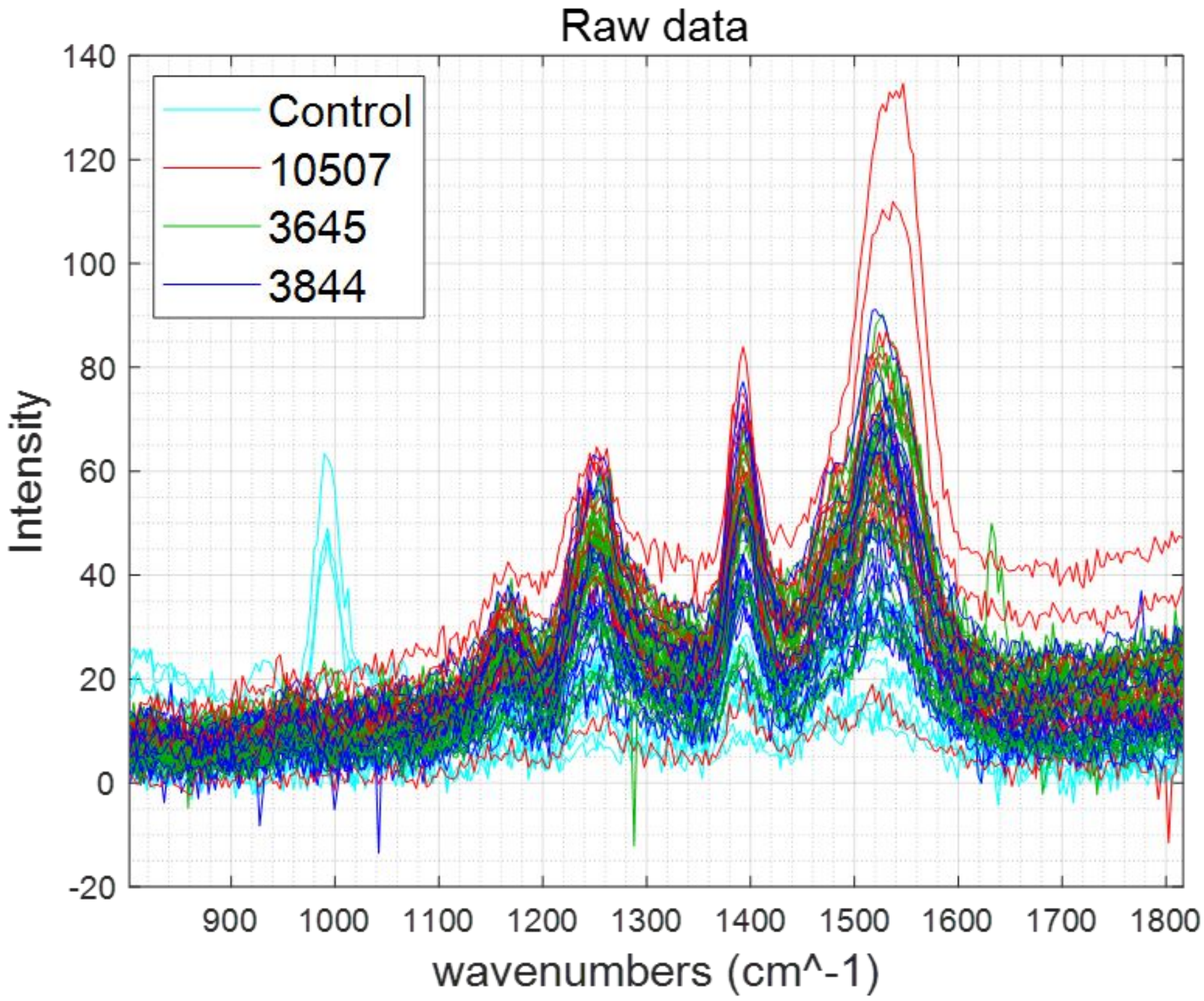

Figure S9: Raw spectra from Raman spectroscopy measurements based on strain.

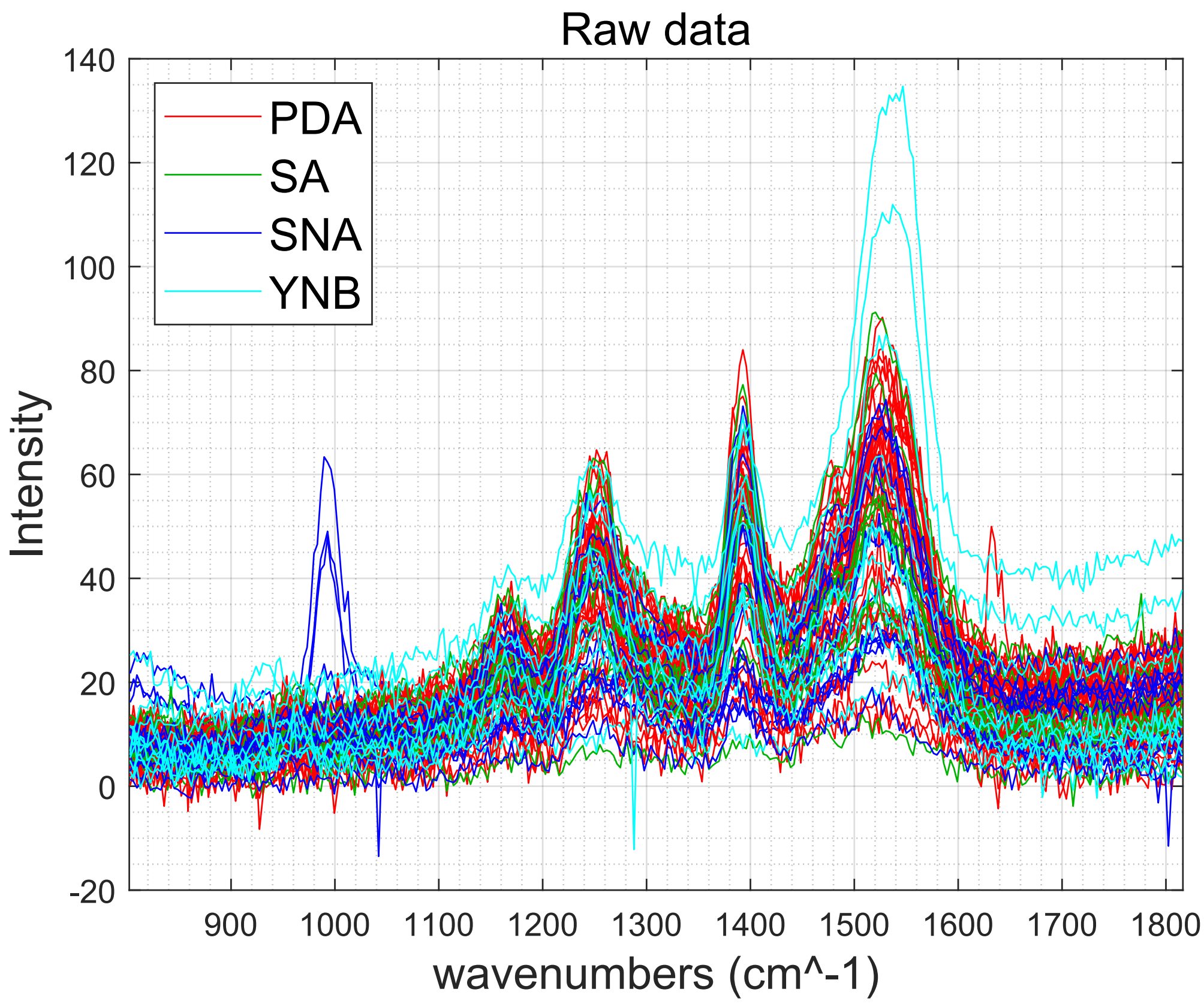

Figure S10: Raw spectra from Raman spectroscopy measurements based on the medium.
